# Design and synthesis of *N*-substituted-2-butyl-4-chloro-1*H*-imidazole derivatives as potential ACE inhibitors: *in vitro* study, *in-silico* analysis, and a multifaceted CSD-supported crystallographic investigation

**DOI:** 10.1039/d5ra04675k

**Published:** 2025-09-24

**Authors:** Manjunath R., Ashwini Rao, Mahesha, Udaya Kumar A. H., Sudarshan Acharya, Padmanabha Udupa E. G., Abdul Ajees Abdul Salam, Sushruta S. Hakkimane, Shashikala B. S., Lokanath N. K., Santosh L. Gaonkar

**Affiliations:** a Department of Chemistry, Manipal Institute of Technology, Manipal Academy of Higher Education Manipal 576104 Karnataka India sl.gaonkar@manipal.edu; b Department of Biochemistry, Kasturba Medical College, Manipal, Manipal Academy of Higher Education Manipal 576104 Karnataka India; c Department of Physics, Sri Jayachamarajendra College of Engineering, JSS Technical Institutions Campus, JSS Science and Technology University Mysuru 570006 Karnataka India; d Department of Physics, Seshadripuram Institute of Technology Mysuru 571311 Karnataka India; e Manipal Institute of Applied Physics, Manipal Academy of Higher Education Manipal 576104 Karnataka India; f Department of Biotechnology, Manipal Institute of Technology Bengaluru, Manipal Academy of Higher Education Manipal 576104 Karnataka India; g Department of Studies in Physics, University of Mysore Manasagangothri Mysuru 570006 Karnataka India

## Abstract

Imidazole derivatives are prominent in medicinal chemistry because of their vast array of biological effects. They are particularly noteworthy in treating hypertension, as demonstrated by imidazole-based medications such as lisinopril and losartan, which are currently available on the market. In this context, six *N*-substituted-2-butyl-4-chloro-1*H*-imidazole derivatives were carefully designed and synthesized through an efficient two-step protocol, with good yields. The synthesized compounds (4a–f) were characterized *via* various analytical techniques, including FTIR, ^1^H NMR, ^13^C NMR, and mass spectrometry. An *in vitro* assessment of angiotensin-converting enzyme inhibition was conducted. The results showed that compound 4b exhibited an exceptional IC_50_ value in the micromolar range (1.31 ± 0.026 μM). Additionally, *in silico* studies were performed, including molecular docking to predict the spatial orientation of the compounds, molecular dynamics simulations to evaluate binding stability with the target protein, and drug likeness studies to ensure adherence to Lipinski's rule. Furthermore, DFT analysis was employed to explore the energy gap of the frontier molecular orbitals (FMOs) and the molecular electrostatic potential (MEP), facilitating the identification of potential nucleophilic and electrophilic attack sites. Comprehensive insights into the molecular structure and packing of compound 4c were obtained through crystallographic studies, Hirshfeld surface analysis, Cambridge Structural Database studies, and energy framework analysis.

## Abstract 2

Imidazole derivatives are prominent in medicinal chemistry because of their vast array of biological effects.

## Graphical abstract

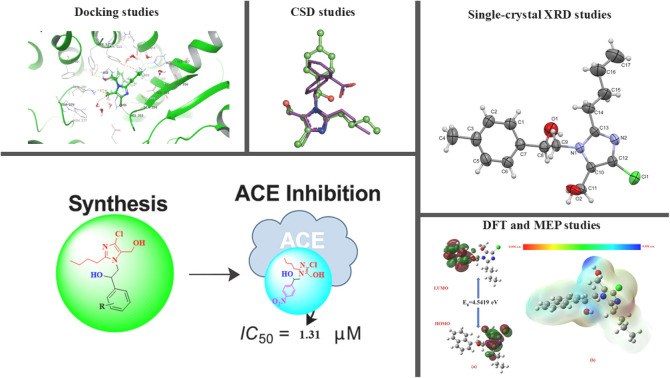

## 1 Introduction

The World Health Organization estimates that nearly 1.13 billion people worldwide have high blood pressure. This condition poses a serious health risk. High blood pressure negatively affects vital organs and dramatically increases the chances of heart disease and stroke.^1,2^ The dipeptide carboxyl metalloproteinase angiotensin-converting enzyme (ACE) plays a crucial role in both the kallikrein-kinin and renin-angiotensin systems. The ACE converts dormant angiotensin I into the vasoconstrictor angiotensin II by cleaving a His–Leu dipeptide.^3^ Bradykinin, a known vasodilator, is inactivated by the ACE enzyme. As a result, inhibiting ACE activity has emerged as a promising approach for reducing blood pressure.^4^ Commonly prescribed ACE inhibitors include captopril, lisinopril, fosinopril, ramipril, and enalapril. While these medications provide therapeutic advantages, their prolonged use leads to adverse effects such as skin rashes, coughing, changes in taste, and potential dysfunction of the liver and kidneys.^5^ Apart from the above-mentioned effects, the current formulations of ACE inhibitors (ACEIs) cause specific side effects such as hyperkalemia, renal failure, and hypotension, particularly following the first dose.^6^ Less frequent but more serious side effects are liver toxicity and angioedema.^7^ Additionally, some studies suggest that ACEIs may lead to tongue angioedema and gingival hyperplasia.^8^ There is also evidence linking adverse fetal reactions to the use of ACEIs.^9^ As a result, there is a pressing need to develop safe, effective, and affordable ACE inhibitors.

**Fig. 1:**
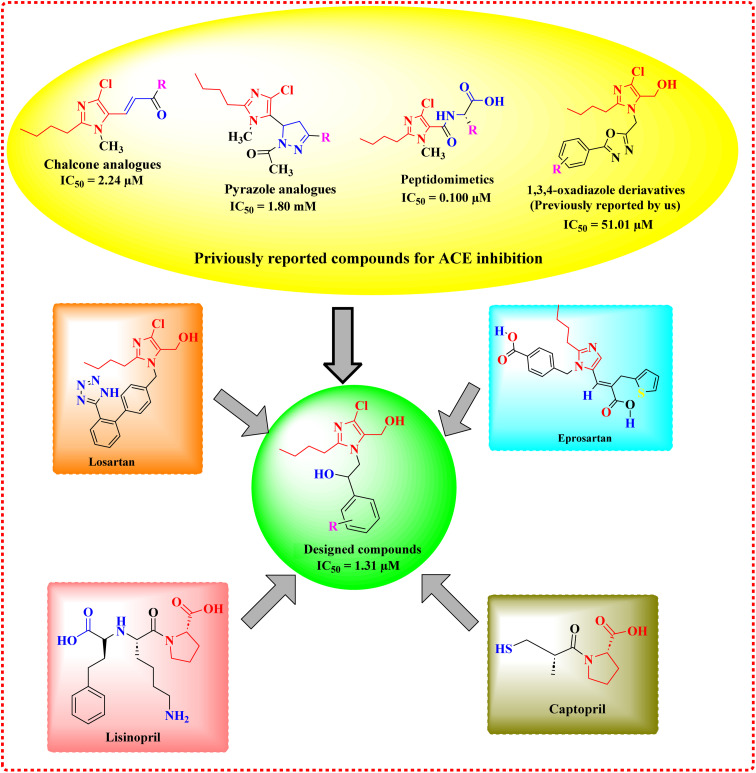
Design strategy.

Clinically, ACE inhibitors are often used in conjunction with medications such as eprosartan, losartan, and other angiotensin II antagonists to manage hypertension and cardiovascular disorders. In pursuit of developing a novel heterocyclic library with drug-like properties for evaluating their efficacy as ACE inhibitors, we incorporated the 2-butyl-4-chloro-1*H*-imidazole unit, derived from the structures of losartan and eprosartan, into a cohesive molecular framework, as illustrated in Fig. 1. The selection of the five-membered, nitrogen-containing substituted imidazole unit was guided by its structural resemblance to the proline unit found in lisinopril and captopril.^10^

In designing the current library, we took account of previously reported scaffolds, including 2-butyl-4-chloro-1*H*-imidazole-derived chalcones (IC_50_ = 2.24 μM) and pyrazole analogues (IC_50_ = 1.80 mM), peptidomimetics (IC_50_ = 0.100 μM), and our previously reported 1,3,4-oxadiazole derivatives (IC_50_ = 51.01 μM), all of which exhibited varying degrees of anti-ACE activity.^11–13^ These comparative insights lead to designing our current compounds, with the goal of enhancing potency and expanding the scope of ACE inhibition.

In our previous work, we synthesized 2-butyl-4-chloroimidazole-derived 1,3,4-oxadiazoles, which exhibited moderate ACE inhibitory activity. In the current study, we adopted a new strategy by reacting 2-butyl-4-chloro-formyl imidazole with phenacyl bromides, followed by the reduction of both the aldehyde and keto groups to yield derivatives with two hydroxyl groups. Unlike chalcones and pyrazoles, which primarily rely on conjugated π-systems for their activity, and peptidomimetics, which mimic natural peptide substrates of ACE but often lack desirable drug-like properties, our current scaffold presents a hybrid design that integrates both lipophilic substituents (butyl, chloro) and polar functionalities (two –OH groups). This balanced combination distinguishes our compounds from previously reported scaffolds and offers a unique framework for investigating ACE inhibition.

This study presents the successful synthesis and evaluation of the ACE inhibitory activity of *N*-substituted-2-butyl-4-chloro-1*H*-imidazole derivatives, accompanied by computational analysis. Additionally, we conducted crystallographic and Hirshfeld studies for compound 4C, along with studies based on the Cambridge Structural Database (CSD).

## 2 Results and discussion

### 2.1 Chemistry

**Scheme 1:**
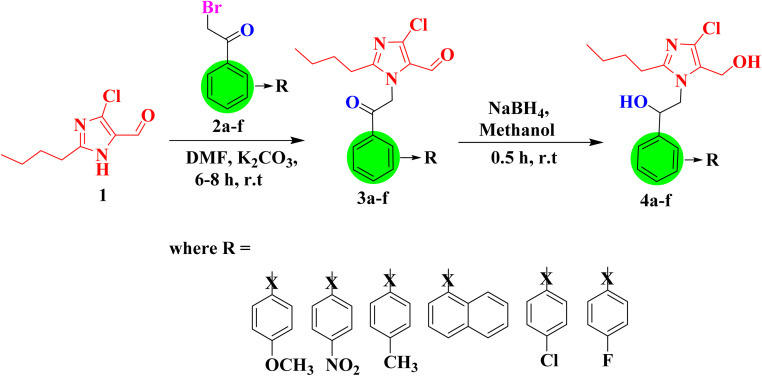
Synthesis of *N*-substituted 2-butyl-4-chloro-1*H*-imidazole derivatives.

In this study, six *N*-substituted-2-butyl-4-chloro-1*H*-imidazole derivatives were synthesized, as shown in Scheme 1. The synthesis process involved the alkylation of 2-butyl-4-chloro-1*H*-imidazole-5-carbaldehyde (1) with different phenacyl bromides (2a–f), resulting in the formation of intermediates (3a–f). These intermediates (3a–f) were subsequently reduced with sodium borohydride to obtain the desired *N*-substituted-2-butyl-4-chloro-1*H*-imidazole derivatives (4a–f).

The FTIR data for the *N*-substituted-2-butyl-4-chloro-1*H*-imidazole derivatives (4a–f) exhibited distinctive bands at various wavenumbers, including 794 cm^−1^ (C–Cl), 1597 cm^−1^ (C=C), 1675 cm^−1^ (C=N), 2833 cm^−1^ (aliphatic C–H), 2954 cm^−1^ (aromatic C–H), and 3181 cm^−1^ (OH). The ^1^H NMR spectra revealed notable signals at *δ* 0.87 for the aliphatic methyl group and peaks at *δ* 1.2 and 1.5 for the aliphatic methylene protons. A signal was detected at *δ* 5.1 for the CH proton, whereas the OH protons appeared at *δ* 5.6 and 5.7. Additionally, aromatic protons were observed in the *δ* 7–8 ppm range. The ^13^C NMR spectrum displayed distinct chemical shifts attributed to the carbons within the imidazole ring, with significant signals at approximately 123, 147, and 148 ppm. The signal for the aliphatic methyl group was at 14.21 ppm, whereas the signals for the aliphatic methylene carbons were at 22.29, 26.16, and 29.42 ppm. Furthermore, a notable signal for CH carbon was found near 71 ppm. The mass of compounds 4a and 4f exhibited peaks at *m*/*z* values of 339.47 and 327.29, respectively.

### 2.2 *In vitro* colorimetric ACE inhibition assay

**Fig. 2:**
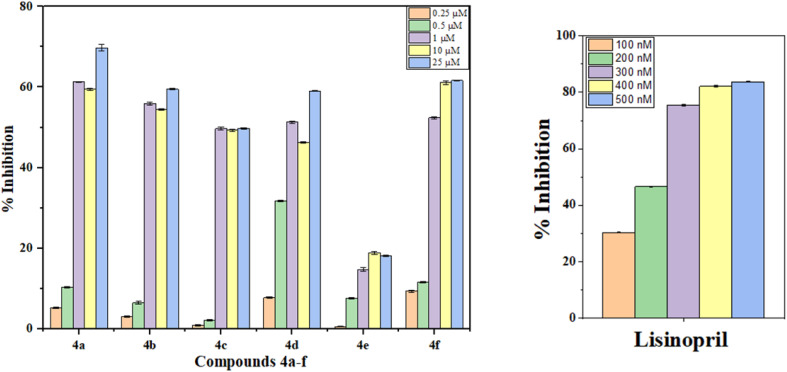
Percent inhibitory effects of compounds 4a–f and lisinopril on the ACE.

The ACE enzyme extract was preincubated with various concentrations of test compounds (4a–f) ranging from 250 nM to 25 μM, alongside the standard lisinopril (100–500 nM), for 10 minutes at 37 °C. The reaction was initiated by adding sodium borate buffer containing 0.3 M sodium chloride and 5 mM HHL, followed by a 30 minutes incubation at 37 °C. To terminate the reaction, 60 μL of 1 M HCl was added, which was subsequently followed by the addition of 120 μL of pyridine and 60 μL of benzene sulfonyl chloride for color development. The mixture was then cooled on ice for 5 minutes, and the resulting yellow color was measured spectrophotometrically at 410 nm. The reduction in HA concentration during the inhibition reaction, compared with that during the control reaction, was expressed as % inhibition, and the IC_50_ values were calculated *via* semilogarithmic plots. The compounds revealed ACE inhibition ranging from 0.59 to 70%. Among the synthesized compounds (4a–f), compound 4b presented the lowest IC_50_ value of 1.31 ± 0.026 μM and the highest enzyme inhibition, whereas compound 4f presented the highest IC_50_ value of 7.57 ± 0.085 μM and the lowest enzyme inhibition. The standard lisinopril had an IC_50_ value of 0.3 ± 0.135 μM. The IC_50_ values for the compounds and the standard lisinopril are presented in Table 1, and a bar graph comprising % inhibition is depicted in Fig. 2.

**Table 1:** IC_50_ values of compounds 4a–f against the ACE

Sr. no	Compound name	IC_50_ (μM)
1	4a	2.47 ± 0.102
2	4b	1.31 ± 0.026
3	4c	1.56 ± 0.065
4	4d	2.12 ± 0.015
5	4e	4.89 ± 0.068
6	4f	7.57 ± 0.085
7	Lisinopril	0.3 ± 0.135

Compounds 4a–f demonstrated superior *in vitro* ACE inhibitory activity compared to previously reported chalcones (IC_50_ = 2.24 μM) and pyrazole analogues (IC_50_ = 1.80 mM), with IC_50_ values ranging from 1.30 to 7.57 μM. Although their potency does not exceed that of peptidomimetics (IC_50_ = 0.100 μM), which are known to mimic the natural substrate of ACE, our compounds present a non-peptidic framework with enhanced drug-like properties. Additionally, the current series shows a significant improvement over our previously reported 1,3,4-oxadiazoles (IC_50_ = 51.01 μM), underscoring the benefits of incorporating two hydroxyl groups alongside lipophilic substituents (butyl and chloro) to achieve an optimal balance between hydrophobic and hydrogen-bonding interactions within the ACE active site. The detailed structure–activity relationships of compounds 4a–f were discussed in Section 2.7.

### 2.3 Single-crystal structure description

**Fig. 3:**
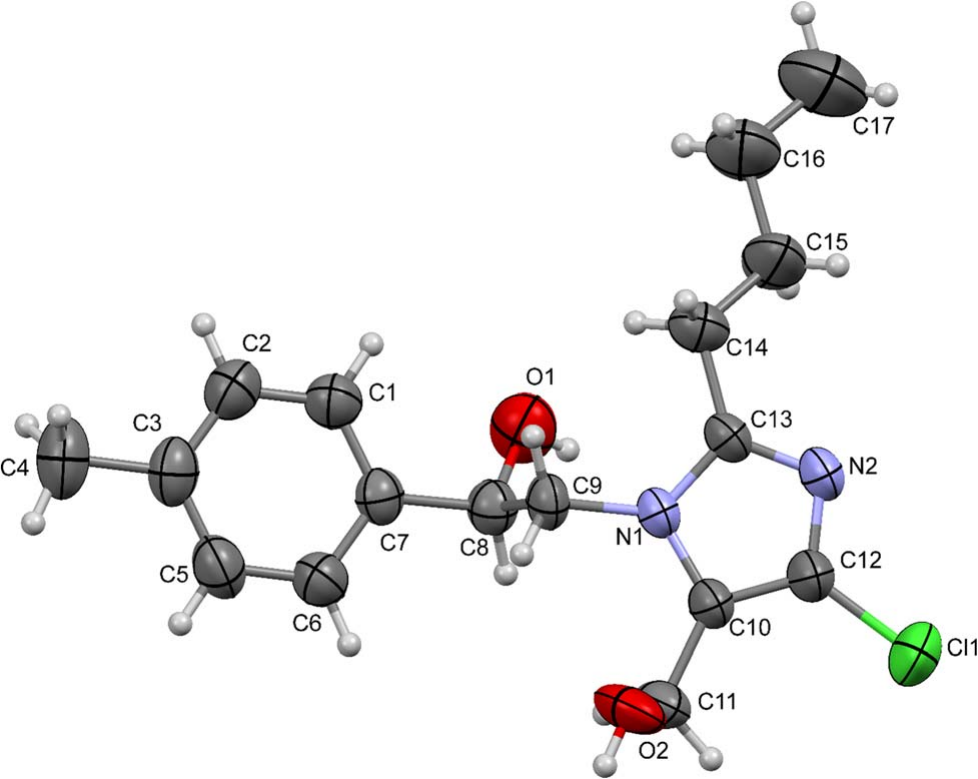
The ORTEP view of compound 4c with thermal ellipsoids was drawn at 50% probability.

Compound 4c is crystallized in a monoclinic crystal system with a *P*2_1_/*c* space group and is summarized in Table 2. The asymmetric unit comprises one symmetry-independent molecule, whereas the unit cell contains four symmetry-dependent molecules (*Z*′ = 1, *Z* = 4). The ORTEP thermal ellipsoid plot, drawn at a 50% probability level, illustrates the molecular structure with an atomic numbering scheme (Fig. 3). The molecular framework comprises two distinct ring systems: the methyl-substituted phenyl ring system (C1/C2/C3/C5/C6/C7) and the five membered ring system (N1/C13/N2/C12/C10). Both the six and five membered rings were analyzed for deviation from planarity, with atomic deviations ranging between −0.006 Å and 0.004 Å. All the atoms in the ring exhibited sp^2^ hybridization, indicating a nearly planar conformation. The phenyl ring has a dihedral angle of 7.63(12)° relative to the five-membered ring, indicating slight distortion and nonplanarity in the molecular structure.

**Table 2:** Details of the single-crystal structure data and refinement parameters of the compound 4c

Parameters	Compound
CCDC deposit no.	2448889
Empirical formula	C_17_H_23_Cl_1_N_2_O_2_
Formula weight	322.837
Temperature (K)	293
Wavelength (Å)	0.71073
Crystal system, space group	Monoclinic, *P*2_1_/*c*
Unit cell dimensions *a* (Å)	11.2517(5)
*b* (Å)	13.8607(6)
*c* (Å)	11.2925(7)
*β* (°)	94.920(5)
Volume (Å^3^)	1754.65(15)
*Z*	4
Density(calculated) (Mg m^−3^)	1.222
Absorption coefficient (mm^−1^)	0.226
*F*_000_	689
Crystal size (mm^3^)	0.22 × 0.19 × 0.23
2*θ* range for data collection	3.64° to 51°
Index ranges	−13 ≤ *h* ≤ 14
	−18 ≤ *k* ≤ 11
	−14 ≤ *l* ≤ 14
Reflections collected	15 638
Unique reflections	3267 [*R*_int_ = 0.0246]
Absorption correction	Multiscan
Refinement method	Full matrix least-squares on *F*^2^
Data/restraints/parameters	3267/55/233
Goodness-of-fit on *F*^2^	1.062
Final [*I* > 2*σ*(*I*)]	*R*_1_ = 0.0521, *wR*_2_ = 0.01319
*R* Indices (all data)	*R*_1_ = 0.0658, *wR*_2_ = 0.1408
Largest diff. Peak and hole	0.22 and −0.41 eÅ^−3^

The structural conformation and crystal packing of the title compound (4c) are stabilized by supramolecular motifs formed through various noncovalent interactions. Intramolecular and intermolecular hydrogen bonds, along with C–H⋯π interactions, play crucial roles in the molecular packing of the structure. The carbonyl O1 atom participates in an intramolecular C–H⋯O interaction with the aromatic H1A atom, forming an S(5) supramolecular pseudo ring. Additionally, the hydroxyl (–OH) group involves an S(6) ring interaction with the aromatic H9A (–CH_2_) atom in the aliphatic chain.

**Fig. 4:**
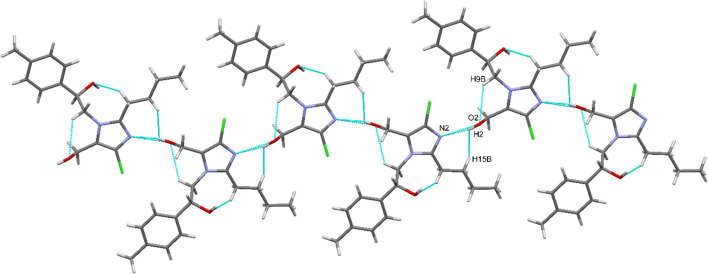
An infinite 1D chain is present in the crystal packing of the molecular fragments along the *b*-axis.

**Fig. 5:**
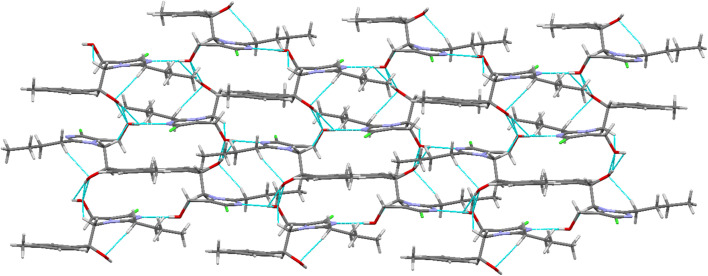
2D planar sheet architecture formed by O–H⋯O hydrogen bonds.

In the crystal lattice, self-assembly is governed by strong, directional intermolecular O–H⋯N hydrogen bonds and dihydrogen (H⋯H) contacts. The carbonyl O1 atom forms an O2–H2⋯N2 hydrogen bond, generating an infinite one-dimensional zigzag chain along the crystallographic *b*-axis (Fig. 4, Table 3). This chain is further reinforced by H2⋯H15B dihydrogen interactions. Notably, the hydroxyl (–OH) group serves as both a hydrogen bond donor and acceptor, interconnecting the 1D chains *via* O–H⋯O interactions, which leads to the formation of a two-dimensional planar sheet, as illustrated in Fig. 4. Fig. 5 further illustrates the 2-D planar sheet architecture formed by the O–H⋯O hydrogen bonds.

**Table 3:** Analysis of the geometry of potential hydrogen bonds of the compound

Type	D–H	H⋯A	D⋯A	D–H…A	Symmetry
C1–H1A⋯O1	0.930(4)	2.418(3)	2.743(3)	100.4(2)	Intra
C1–H1B⋯O3	0.970(3)	2.328(8)	3.067(8)	132.4(3)	Intra
C14–H14A⋯O1	0.970(8)	2.376(5)	3.190(5)	141.2(5)	Intra
O1–H1⋯O2	0.818(13)	1.881(15)	2.673(3)	163(3)	*x*, 1/2 − *y*, −1/2 + *z*
O1–H2⋯N2	0.822(14)	1.967(12)	2.777(2)	168(2)	1 − *x*, −1/2 + *y*, 3/2 − *z*

Furthermore, weak C–H⋯π interactions offer additional stabilization for crystal packing. Specifically, the C4–H4B⋯π and C14–H14B⋯π interactions facilitate parallel stacking with adjacent molecules located at symmetric positions (−*x*, −*y*, 1 − *z*) and (*x*, 1/2 − *y*, 1/2 + *z*), respectively, reinforcing the total stability of the crystal structure (Fig. S25 provided in the SI Material).

### 2.4 Hirshfeld surface analysis

#### 2.4.1 *d*_norm_ and shape index surface analysis

**Fig. 6:**
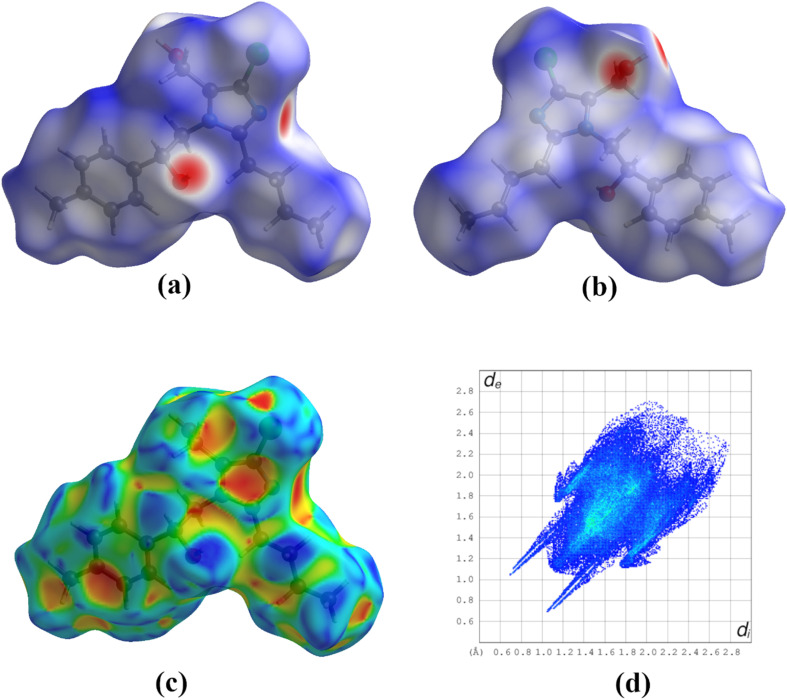
(a) and (b) Hirshfeld surface of the compound mapped over the *d*_norm_ surface, (c) shape index mapped surface, (d) 2D fingerprint plots of the compound.

The noncovalent intermolecular interactions in the crystalline phase were quantitatively analyzed using the Hirshfeld surface. The Hirshfeld surface mapped over *d*_norm_ and shape index properties are shown in Fig. 6. On the *d*_norm_ surface, a red-colored area near the nitrogen atom of the five-membered ring specifies O–H⋯N interactions, which contribute to the formation of the 1D chain. Two vivid red-colored regions on the oxygen and hydrogen atoms of the hydroxyl group (Fig. 6a and b) indicate the propensity of the –OH moiety, which acts as both a donor and acceptor, forming two O–H⋯O hydrogen bonds that establish a 2D packing arrangement.^14^

In the shape index surface, a blue-colored bulged area near the –CH atom of the phenyl ring and the –CH_2_ group, along with a red-colored dip on the ring system, corresponds to the weak C–H⋯π interactions seen in the crystal packing analysis (Fig. 6c). These interactions further contribute to the total stabilization of the molecular assembly in the crystal lattice.

#### 2.4.2 Fingerprint and enrichment ratio analysis

The fingerprint plots derived from the Hirshfeld surface provide a quantitative scrutiny of intermolecular interactions within the crystal. The percentage contribution of each contact type was obtained from decomposed 2D fingerprint plots (Fig. 6d). Among these interactions, H⋯H contacts dominate the crystal packing, contributing 55.4% to the total Hirshfeld surface. This is evident from the characteristic spike near *d*_e_ ≈ *d*_i_ ≈ 1.2 Å, indicating close hydrogen–hydrogen interactions.

Additionally, the horn-shaped symmetrical pattern observed at *d*_e_ + *d*_i_ ≈ 3.0 Å corresponds to H⋯Cl contacts, accounting for 14.8% of the Hirshfeld surface. The presence of C⋯H interactions, attributed to weak C–H⋯π interactions, is marked by two symmetrical wings at *d*_e_ + *d*_i_ ≈ 2.8 Å, contributing 14.1% of the total surface area. Notably, two symmetrical wings observed at *d*_e_ + *d*_i_ ≈ 2.9 Å in the (*d*_e_, d_i_) bin correspond to H⋯O/O⋯H contacts, contributing 7.3% and confirming the presence of O–H⋯O hydrogen bonds, as previously identified in the structural analysis.

Favored and disfavored contacts from a chemical element point of view can be emphasized *via* the enrichment ratios, as shown in Table 4. The di-hydrogen interaction (*E*_HH_ = 0.94) is slightly underrepresented, indicating a minor tendency for self-interaction. An *E*_CH_ value of 1.12 clearly indicates favorable interactions, likely due to aromatic C–H⋯π stacking interactions. N–N interactions are avoided, whereas N–H contacts are enriched because of hydrogen bonding (*E*_NH_ = 1.18). Oxygen prevents self-interaction but favors hydrogen bonding *via* O–H⋯O interactions (*E*_OH_ = 1.25). Overall, hydrogen bonding (O–H, N–H, and C–H) remains a key interaction mechanism in the crystal packing of a given organic structure.^15^

**Table 4:** Actual contact from the Hirshfeld surface and derived random contact and enrichment ratios for compound 4c^a^

	H	C	N	O	Cl
H	55.4			Actual contacts	
C	14.1	0.9			
N	6.8	0.4	0		
O	7.3	0	0.3	0	
Cl	14.8	0	0	0	0
Sx	76.9	8.15	3.75	3.8	7.4
H	59.14			Random contacts	
C	12.53	0.66			
N	5.77	0.61	0.14		
O	5.84	0.62	0.29	0.14	
Cl	11.38	1.21	0.56	0.56	0.55
H	0.94			Enrichment ratio	
C	1.12	—			
N	1.18	—	—		
O	1.25	—	—	—	
Cl	1.30	0.00	—	—	—

a (The enrichment ratios were not computed when the ‘random contacts’ were lower than 0.9%, as they are not meaningful.).

#### 2.4.3 Interaction energy and energy framework analysis

**Fig. 7:**
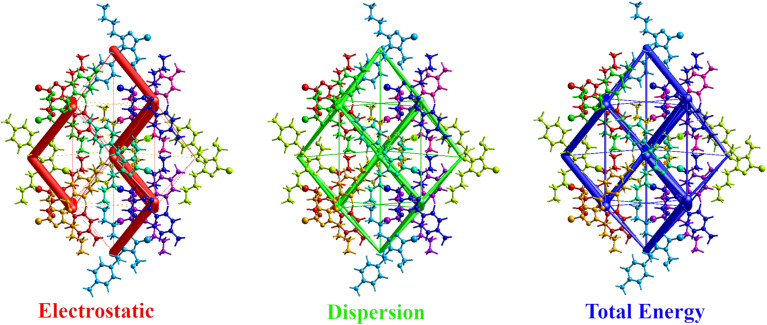
The energy components of the molecular fragments are based on their interaction energies.

Energy framework analysis provides insights into the three-dimensional topology of molecular interactions by computing electrostatic (*E*_ele_), polarization (*E*_pol_), dispersion (*E*_dis_), repulsion (*E*_rep_), and total interaction (*E*_tot_) energies. Table 5 below summarizes the computed interaction energy components for the title compound (4c). The obtained energy values are plotted and visualized through an energy framework that consists of electrostatic (*E*_ele_ = −150.5 kJ mol^−1^: represents the stabilizing interactions due to charge distribution), dispersion energy (*E*_dis_ = 184.5 kJ mol^−1^: represents van der Waals forces contributing to molecular cohesion), and total interaction energy (*E*_tot_ = −240.3 kJ mol^−1^: provides an overall visualization of the dominant forces holding the structure together). The energy frameworks illustrate the anisotropic nature of intermolecular interactions, showing that electrostatic and dispersion forces are crucial in stabilizing the crystal packing (Fig. 7 and Table 5). The strongest interaction is found for the (*x*, −*y* + 1/2, *z* + 1/2) symmetry-related molecule at 5.73 Å, with a total interaction energy of −71.1 kJ mol^−1^, indicating a highly stabilizing effect. The strongest stabilizing interactions arise from a combination of electrostatic and dispersion energies.^16^

**Table 5:** Interaction energies (kJ mol^−1^) between the molecular fragments

	N	Symop	*R*	*E*_ele_	*E*_pol_	*E*_dis_	*E*_rep_	*E*_tot_
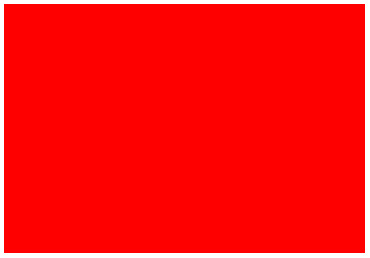	2	−*x*, *y* + 1/2, −*z* + 1/2	11.68	−1.2	−0.3	−16.1	7.5	−10.9
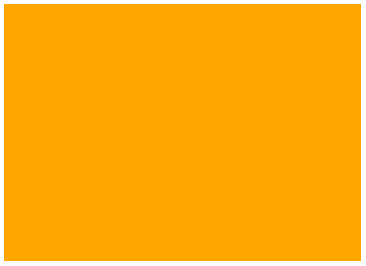	1	−*x*, −*y*, −*z*	10.60	−4.4	−0.6	−34.5	17.8	−24.2
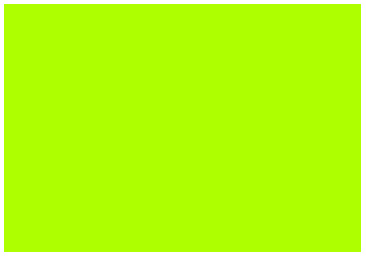	2	*x*, −*y* + 1/2, *z* + 1/2	12.19	1	−0.4	−5.6	0	−4.1
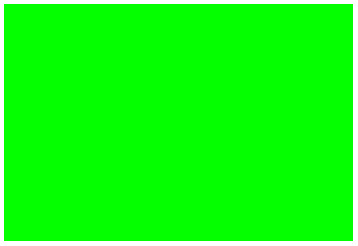	1	−*x*, −*y*, −*z*	9.28	−1.7	−0.1	−24.1	7.8	−18.1
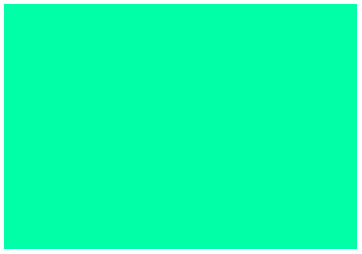	2	*x*, −*y* + 1/2, *z* + 1/2	5.73	−57.4	−12.9	−53.5	73.8	−71.1
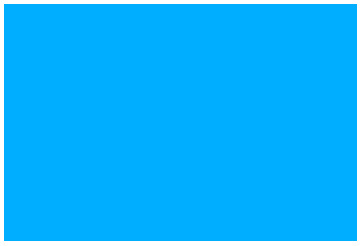	2	*x*, *y*, *z*	13.86	−2.9	−0.3	−9	0	−11.1
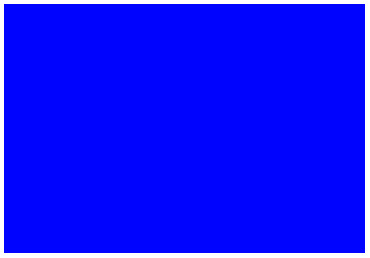	2	−*x*, *y* + 1/2, −*z* + 1/2	9.07	−59.9	−16.1	−28	79.7	−50.5
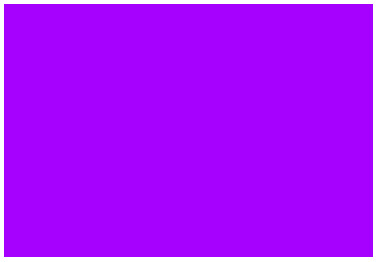	1	−*x*, −*y*, −*z*	9.03	0.8	−1.3	−11.7	1.9	−9.2
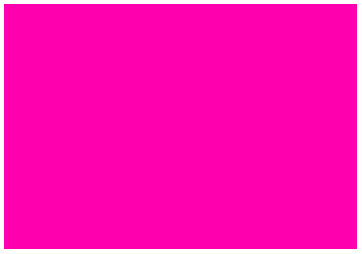	1	−*x*, −*y*, −*z*	7.43	−16.7	−4.2	−29.4	8.2	−41.4
				**−150.517**	**−26.788**	**−184.565**	**121.5606**	**−240.309**

### 2.5 Cambridge structural database (CSD) analysis

**Fig. 8:**
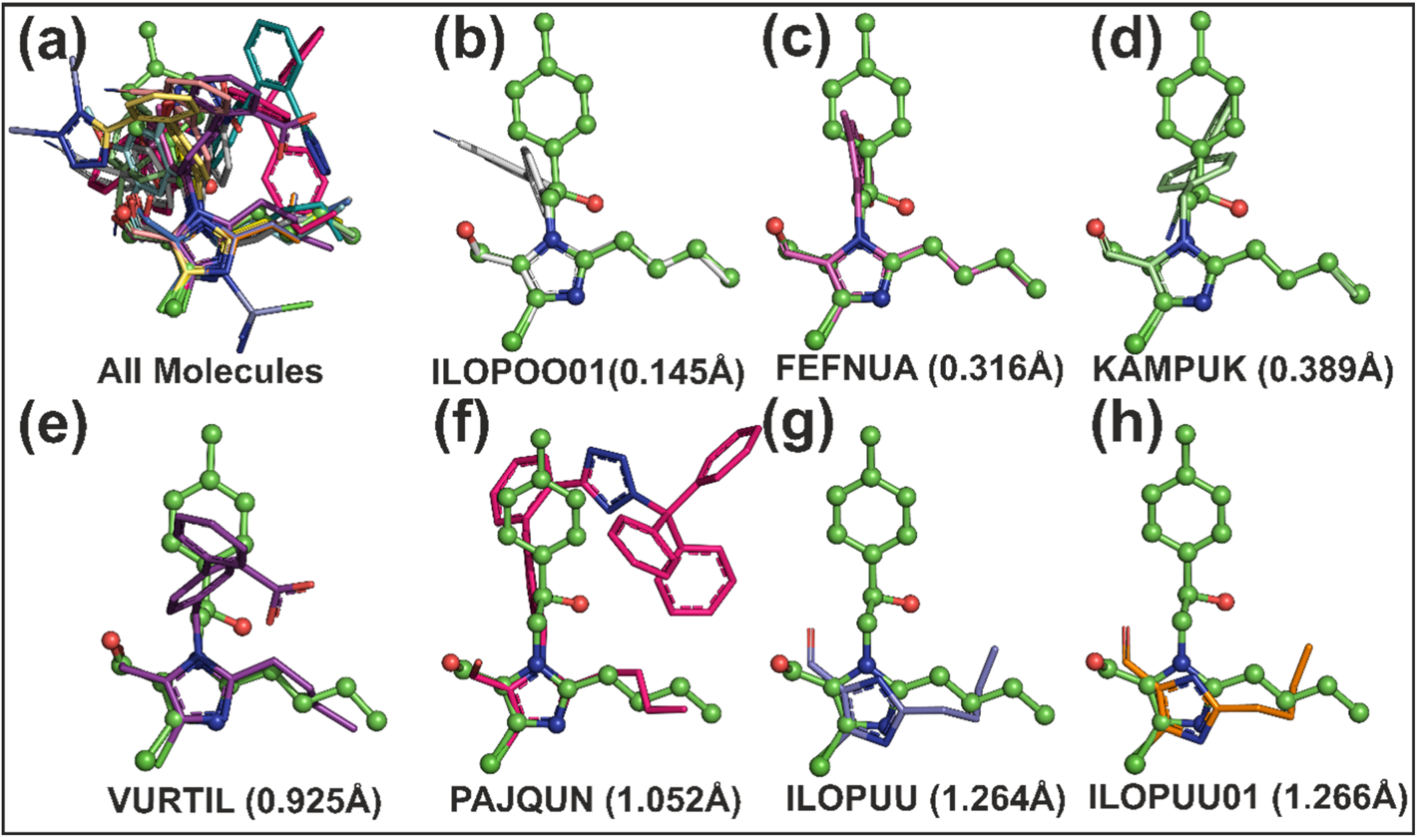
Superposition of CSD molecules on compound 4c. (a) Fourteen CSD molecules superimposed on 4c; (b), (c), and (d) show molecules with the lowest RMSD values; (e), (f), (g), and (h) show molecules with the highest RMSD deviations.

To investigate the structural similarity and conformational variability of molecule 4c, a comparative analysis was conducted using 14 molecular structures retrieved from the CSD. These CSD entries were selected on the basis of the presence of a common substructure corresponding to the (2-butyl-4-chloro-1*H*-imidazol-5-yl)methanol framework, which served as the basis for molecular superimposition. A 12-atom core representing this shared skeleton was employed to align all the structures onto the conformation of molecule 4c (Fig. 8a). The root mean square deviation (RMSD) values among the superimposed structures ranged from 0.145 Å to 1.266 Å, indicating varying degrees of conformational congruence. The lowest RMSD value was observed for CSD entry ILOPOO01, reflecting a high degree of structural similarity with 4c and minimal deviation in atomic positions within the aligned core (Fig. 8b). In contrast, the highest RMSD value of 1.266 Å, recorded for ILOPOUU01, points to more pronounced conformational differences (Fig. 8h). Other entries with comparatively elevated RMSD values included YALXOZ (0.913 Å), VURTIL (0.925 Å), PAJQUN (1.052 Å), and ILOPOU (1.264 Å). These deviations are attributed primarily to flexibility within the terminal methanol/formaldehyde group and the butyl side chain, both of which are capable of adopting multiple rotamers or orientations owing to the absence of strong steric or electronic constraints. To further elucidate the origins of these structural discrepancies, a detailed analysis of torsion angles within these flexible moieties was performed.

**Fig. 9:**
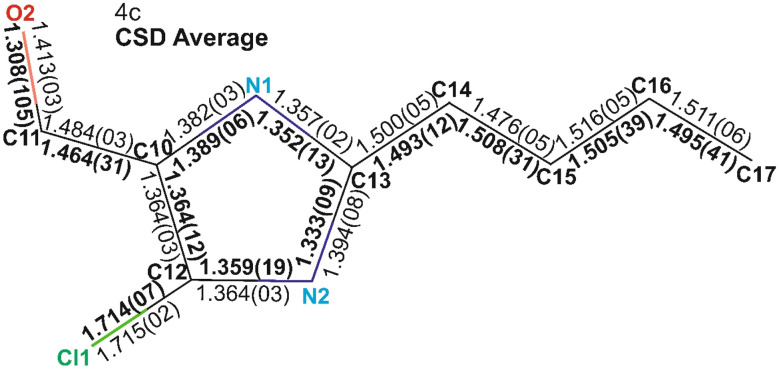
Comparative analysis of bond lengths between molecule 4c and CSD structures. (A) Comparison of the bond lengths between molecule 4c and the average bond lengths derived from the corresponding CSD structures. The bond lengths for 4c are shown in regular text, whereas the corresponding average values from the CSD structures are displayed in bold black font.

**Fig. 10:**
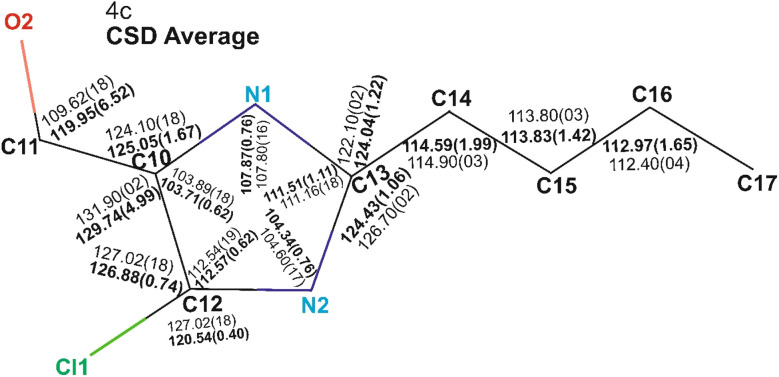
Comparative analysis of bond angles between molecule 4c and CSD structures. (A) Comparison of the bond angles between molecule 4c and the average bond angles derived from the corresponding CSD structures. The bond angles for 4c are shown in regular text, whereas the corresponding average values from the CSD structures are displayed in bold black font.

To further evaluate structural consistency, the bond lengths and bond angles of molecule 4c were compared with the average values derived from 14 CSD structures sharing the common (2-butyl-4-chloro-1*H*-imidazol-5-yl)methanol framework. Overall, the bond lengths and angles in 4c were largely consistent with the CSD averages, with most values falling within the respective standard deviations. However, a notable deviation was observed in the C11–O2 bond length, which differed significantly from the mean (Fig. 9 and SI Table 1). This deviation can be attributed to structural variations among the CSD entries. Specifically, for the molecules QAKXAF, YALXOZ, PAJQUN, ILOPOO, OCAHAC, and VURTIL, the C11–O2 bond length ranges from 1.353 Å to 1.440 Å. These structures predominantly feature a methanol group as the terminal side chain, except for VURTIL, which contains a formaldehyde group. In contrast, the remaining CSD structures that possess a formaldehyde moiety exhibit shorter C11–O2 bond lengths, typically in the range of 1.193 Å to 1.219 Å. The elongation of the C–O bond in methanol-containing structures can be attributed to the electron-donating hydroxyl group, which introduces greater electron density and potential hydrogen bonding interaction factors known to lengthen the C–O bond.^17,18^ Similarly, significant variation was observed in the C10–C11–O2 bond angle, with a standard deviation of 6.52° across the 14 CSD structures (Fig. 10 and SI Table 2). For molecules such as VURTIL, ILOPOO, YALXOZ, OCAHAC, PAJQUN, QAKXAF, and ILOPOO01, the bond angles ranged from 111.79° to 115.77°. These molecules primarily contain a methanol terminal group, except for VURTIL. In contrast, structures with a formaldehyde side chain displayed larger C10–C11–O2 bond angles, varying from 124.10° to 127.74°. The discrepancy in bond length and angle for VURTIL, despite its formaldehyde moiety, may be influenced by π–π stacking interactions within the crystal lattice, which can locally alter the molecular conformation and geometry.^19^

The torsion angles were measured for both the methanol/formaldehyde side chains and the butyl chains across all 14 CSD structures (SI Table 3). The methanol side chains in molecules such as ILOPOO, YALXOZ, OCAHAC, PAJQUN, QAKXAF, and ILOPOO01 predominantly adopt a ± sc ± ac (synclinal/anticlinal) conformation, and molecule 4c follows the same pattern. In contrast, the formaldehyde side chains in molecules FEFNOU, FEFNUA, HAMRET, ILOPUU, ILOPUU01, KAMPUK, and WAKJEY exhibit a ± sp ± ap (synperiplanar/antiperiplanar) conformation. Interestingly, the formaldehyde side chain of VURTIL deviates from this trend and adopts a ± sc ± ac conformation, similar to that observed in methanol-bearing molecules. This deviation aligns with the bond length and bond angle anomalies observed for VURTIL, further suggesting that its geometry may be influenced by local structural factors, such as π–π stacking interactions within the crystal lattice. In the case of the butyl side chains, the molecules ILOPOO, ILOPOO01, FEFNUA, and KAMPUK adopt a ±ap ± sp ± ap ± ap (antiperiplanar/synperiplanar/antiperiplanar/antiperiplanar) conformation, a pattern that is also observed in molecule 4c. In contrast, the butyl chain of FEFNOU exhibited a ±ap ± sp ± ap–ac conformation, whereas VURTIL adopted a ±ap ± sp + sc ± ap arrangement. The HAMRET and QAKXAF molecules both display an -ac + sc ± ap ± ap conformation. ILOPUU and ILOPUU01 share a −ac + sc + sc + sc configuration, whereas OCAHAC adopts a +ac–ac ± ap–sc conformation. PAJQUN displays a +sc–ac + sc ± ap conformation, and both WAKJEY and YALXOZ exhibit a +sc-ac ± ap ± ap pattern. These diverse torsional conformations reflect the sensitivity of the butyl chains to local intramolecular and intermolecular environments. In particular, C–H⋯π interactions appear to be a key factor influencing the observed conformational variability of the butyl moieties.

### 2.6 Computational studies

#### 2.6.1 DFT studies

DFT calculations offer significant insights into the electronic structure, reactivity, and stability of molecules.

The optimized energies of compounds 4a–f are depicted in Table 6, which indicates their relative stability. Among all the structures, 4f has the highest optimized energy (−1802.380 hartree), suggesting that it is the most stable configuration. Conversely, 4c has the lowest optimized energy (−1382.094 hartree), implying that it is the least stable among the given structures.

**Table 6:** Chemical reactive parameters of the compounds (4a–f)

Name	4a	4b	4c	4d	4e	4f
Optimized energy	−1457.322	−1547.329	−1382.094	−1496.437	−1442.034	−1802.380
*E*_homo_ (hartree)	−0.22789	−0.2392	−0.2291	−0.22968	−0.23295	−0.23345
*E*_lumo_ (hartree)	−0.02612	−0.10967	−0.02688	−0.06277	−0.03803	−0.04009
*E*_homo_ (eV)	−6.2012	−6.5090	−6.2341	−6.2499	−6.3389	−6.3525
*E*_lumo_ (eV)	−0.7108	−2.9843	−0.7314	−1.7081	−1.0348	−1.0909
Energy gap	5.4904	3.5247	5.5022	4.5419	5.3040	5.2616
Ionization energy (*I*)	6.2012	6.5090	6.2341	6.2499	6.3389	6.3525
Electron affinity (*A*)	0.7108	2.9843	0.7314	1.7081	1.0348	1.0909
Electronegativity(*χ*)	3.4560	4.7466	3.4828	3.9790	3.6869	3.7217
Chemical potential (*μ*)	−3.4560	−4.7466	−3.4828	−3.9790	−3.6869	−3.7217
Global hardness (*η*)	2.7452	1.7623	2.7513	2.2709	2.6520	2.6308
Global softness (*s*)	0.3643	0.5674	0.3635	0.4403	0.3771	0.3801
Electrophilicity index (*ω*)	2.1754	6.3922	2.2043	3.4859	2.5628	2.6325

The highest occupied molecular orbital (HOMO) electron density is largely concentrated on the phenyl ring and hydroxyl (–OH) groups, highlighting their strong electron-donating nature. This localization suggests that these regions are highly reactive toward electrophilic attack. In contrast, the lowest unoccupied molecular orbital (LUMO) is distributed mainly over electronegative atoms such as nitrogen and oxygen, indicating their susceptibility to nucleophilic interactions. The charge separation between the HOMO and LUMO underscores a significant charge transfer capability, further supporting the observed stability and reactivity trends of the molecule.

**Fig. 11:**
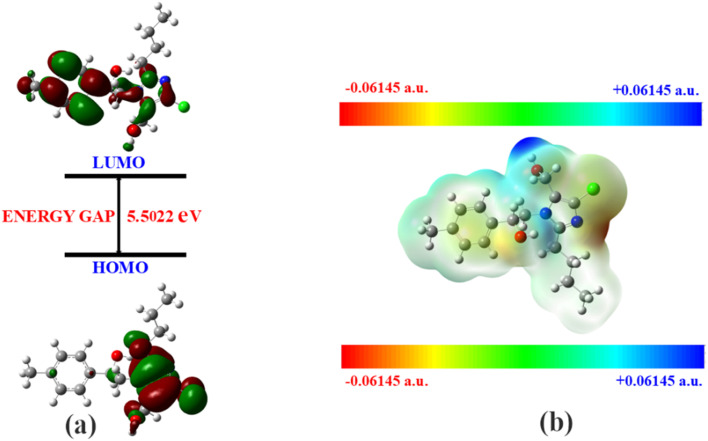
(a) FMOs along with the Δ*E* of 4c and (b) MEP of the molecule.

**Fig. 12:**
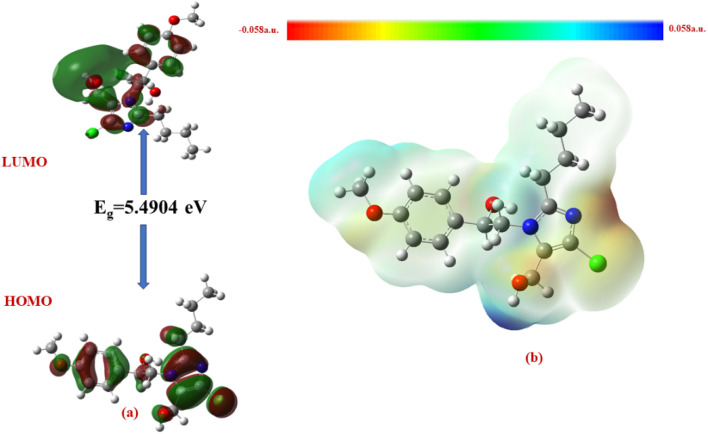
(a) FMOs along with the Δ*E* of 4a, (b) MEP of the molecule.

**Fig. 13:**
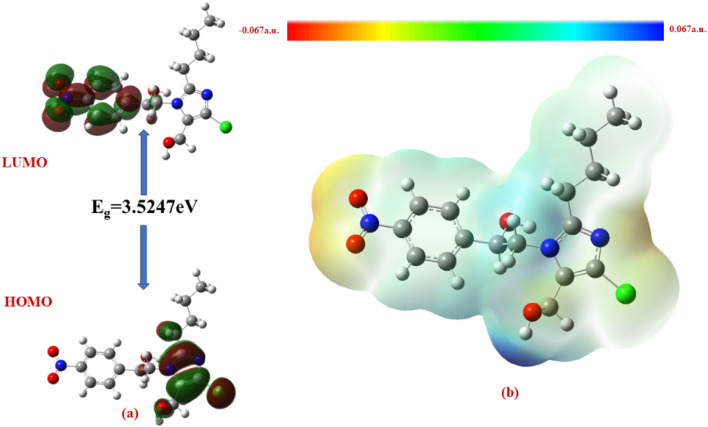
(a) FMOs along with the Δ*E* of 4b, (b) MEP of the molecule.

**Fig. 14:**
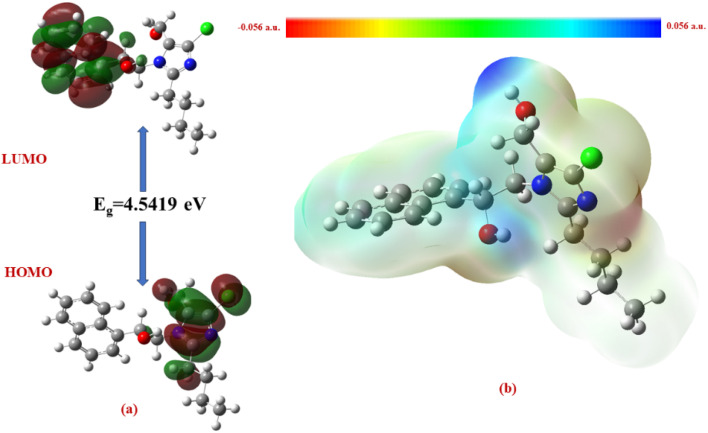
(a) FMOs along with the Δ*E* of 4d and (b) MEP of the molecule.

**Fig. 15:**
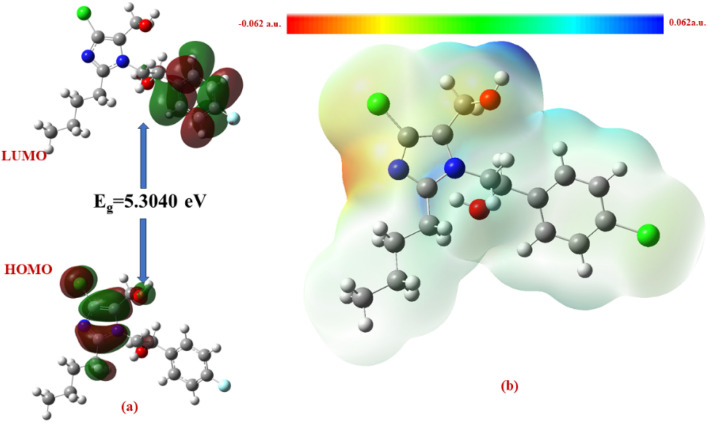
(a) FMOs along with the Δ*E* of 4e, (b) MEP of the molecule.

**Fig. 16:**
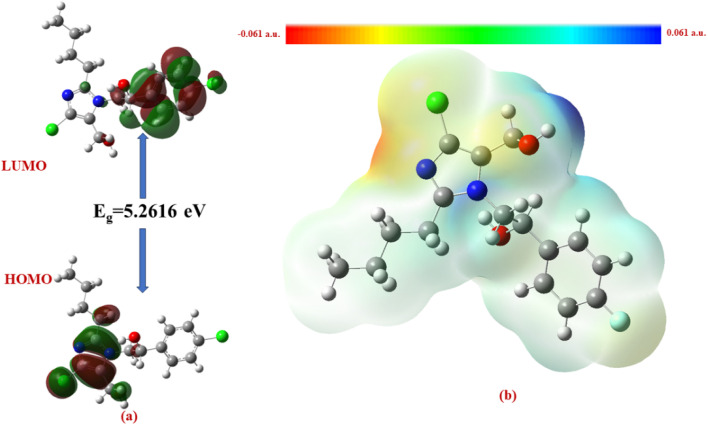
(a) FMOs along with the Δ*E* of 4f and (b) MEP of the molecule.

The Δ*E* values of the FMOs of compounds 4a–f are depicted in Figs. 11(a) to 16(a). The wide HOMO–LUMO energy gap signifies high chemical stability and low reactivity. Additionally, the molecular electronegativity (*χ*) suggests a tendency to attract and donate electrons. The high ionization energy (*I*) reinforces the molecule's stability, whereas the electrophilicity index (*ω*) specifies a potential to accept electrons in chemical interactions.

The HOMO–LUMO energy gap (Δ*E*) manifests the electronic stability and reactivity. 4a (5.4904 eV), 4c (5.5022 eV), 4e (5.3040 eV), and 4f (5.2616 eV) have relatively large energy gaps, suggesting greater stability and lower chemical reactivity. On the other hand, 4b (3.5247 eV) has the smallest energy gap, indicating higher reactivity due to its greater electron acceptor–donor interaction potential.

The ionization energy (*I*) and electron affinity (*A*) values provide insights into the ability of these compounds to donate or accept electrons. 4b has the highest electron affinity (2.9843 eV), making it the most electrophilic and highly reactive among the given structures. The electronegativity (*χ*) trend follows a similar pattern, with 4b having the highest value (4.7466 eV), indicating its strong tendency to attract electrons. The chemical potential (*μ*) values indicate that 4b (−4.7466 eV) is the most reactive, whereas 4c (−3.4828 eV) is the least reactive.

The global hardness (*η*) and global softness (*s*) parameters indicate the resistance to electron transfer. 4a, 4c, 4e, and 4f exhibit higher hardness values (>2.6 eV), confirming their stability, whereas 4b (1.7623 eV) is the softest and most reactive. The electrophilicity index (*ω*) further supports this trend, with 4b having the highest value (6.3922 eV), suggesting that it is the most electrophilic and prone to reactions. The whole chemical reactive parameters of compounds (4a–f) are provided in Table 6.

From the MEP analysis (Fig. 11(b)–16(b)), the regions exhibiting negative electrostatic potential correspond to sites susceptible to nucleophilic attack, which are predominantly located on the hydroxyl groups and nitrogen atoms. These areas demonstrate a strong propensity for electron donation during chemical reactions. The hydroxyl group (−0.03724 a.u.) and the phenyl ring (−0.01467 a.u.) also display moderate nucleophilicity, indicating their potential role in charge transfer processes. On the other hand, the electrophilic sites, indicated by positive electrostatic potential values, are concentrated on the –CH and –CH_2_ groups (0.01885 and 0.02190 a.u.) as well as the hydroxyl (–OH) groups (0.03740 a.u). These regions serve as electron-deficient centers, making them more prone to nucleophilic attack. The MEP visualization confirms that the molecular surface has a well-defined electrostatic distribution, which influences intermolecular interactions, including hydrogen bonding and charge transfer reactions.

In all the compounds, the –OH group is red in color, indicating a high electron density region, making it a potential site for electrophilic attack. Conversely, the nitrogen atom in the five-membered ring appears blue, indicating a low electron density region, which suggests its tendency to attract electrons and act as an electrophile.

Additionally, the O–CH_3_ group is yellow in color, indicating moderate electron density, which may contribute to electron-donating or electron-withdrawing effects depending on the molecular environment. The C–H group appears light blue, suggesting slightly electron-deficient regions, making it less reactive than the other functional groups.

A unique feature observed in the MEP map is that halogens exhibit dual-color behavior, indicating an anisotropic charge distribution. This means that halogens can function as both nucleophiles and electrophiles, depending on their molecular interactions and external influences.

#### 2.6.2 Molecular docking

**Fig. 17:**
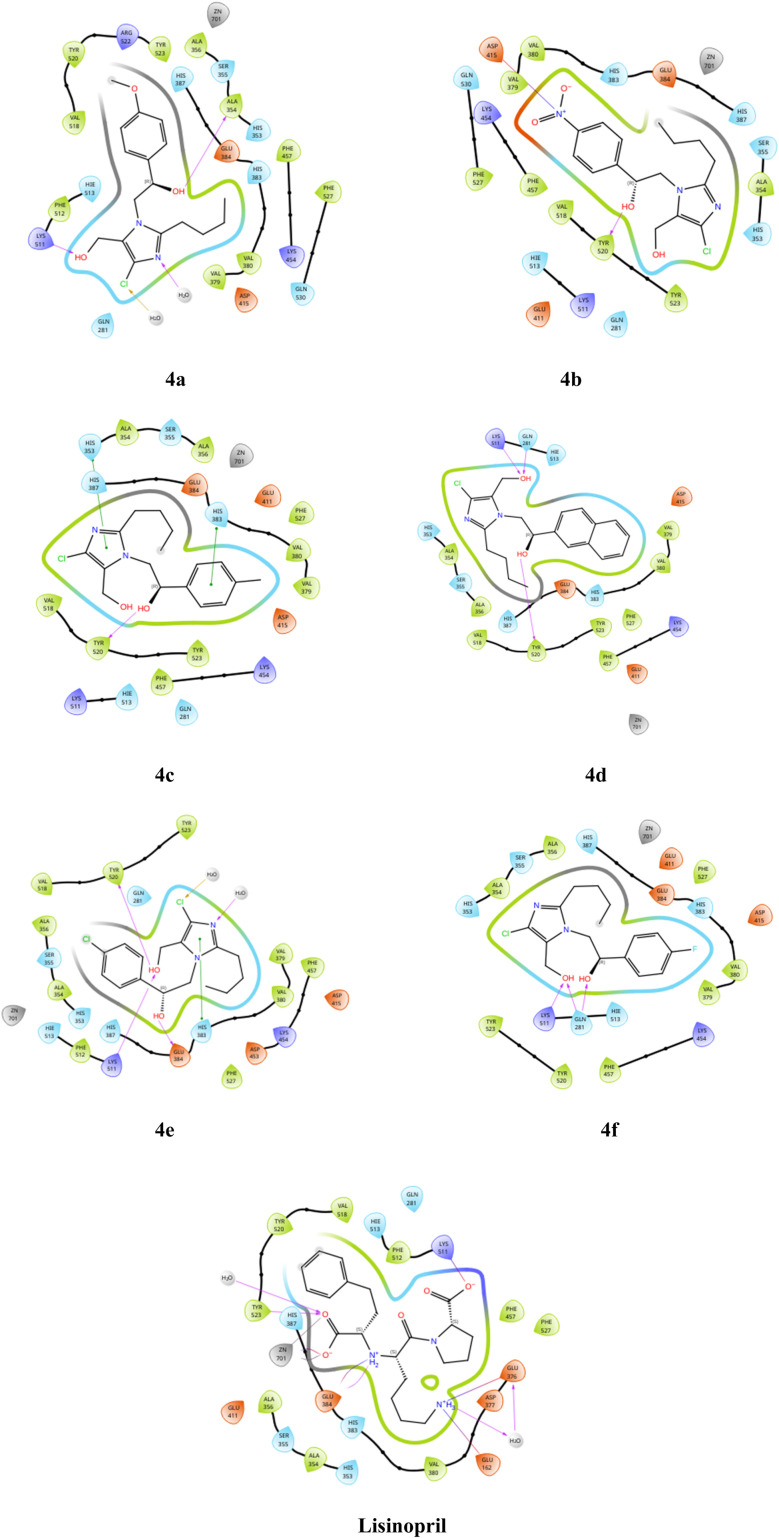
Docking poses of compounds (4a–f).

To gain a deeper understanding of how ligands interact with proteins, we performed docking simulations using the Glide module of Schrödinger 2024-3.^20^ The protein chosen for the docking study was sourced from the Protein Data Bank and is accessible through the RCSB PDB repository. Specifically, we focused on the human angiotensin-converting enzyme in complex with lisinopril (PDB ID: 1O86).^13,21^Table 7 below illustrates all the possible interactions between the compounds (4a–f) and the standard lisinopril with various amino acid residues of the protein. The 2d docking poses of compounds (4a–f) are shown in Fig. 17.

**Table 7:** Docking score, glide score, and various interactions of compounds (4a–f)

Sl. no	Compound name	Docking score	Glide score	Interactions
1	4a	−5.161	−5.168	H-bond: ALA 354, and LYS 511
				Polar: HIE 513, GLN 281, HIS 383, HIS 387
				Halogen: H_2_O
				Hydrophobic: VAL 379, VAL 380, PHE 457, PHE 527
2	4b	−5.508	−5.515	H-bond: TYR 520
				Salt bridge: ASP 415
				Polar: HIE 513, GLN 281, HIS 353, HIS 383
				Hydrophobic: VAL 380, VAL 379, PHE 457, VAL 518, TYR 523
3	4c	−5.826	−5.833	H-bond: TYR 520
				Pi–Pi stacking: HIS 383, HIS 387, HIS 353
				Hydrophobic: VAL 518, TYR 523, VAL 379, VAL 380, and PHE 527
4	4d	−6.311	−6.318	H-bond: LYS 511, GLN 281, and TYR 520
				Hydrophobic: ALA 354, ALA 356, VAL 380, and VAL 379
5	4e	−5.177	−5.185	H-bond: TYR 520, LYS 511, GLU 384
				Halogen: H_2_O
				Pi–Pi stacking: HIS 383
				Hydrophobic: ALA 354, VAL 518, ALA356, TYR 523, VAL 379, VAL 380, PHE 457, PHE 527
6	4f	−6.218	−6.226	H-bond: LYS 511 and GLN 281
				Polar: HIE 513, HIS 383
				Hydrophobic: ALA 354, ALA 356, TYR 523, PHE 527
7	Lisinopril	−14.284	−14.417	H-bond: GLU 376, GLU 162, TYR 523, GLU 384
				Salt bridge: LYS 511
				Polar: HIE 513, GLN 281, HIS 387
				Hydrophobic: VAL 518, TYR 520, PHE 512, PHE 457, PHE 527, VAL 380
				Metal coordination: Zn-701

#### 2.6.3 MD simulation

The stability of the docked structures of 4b, 4c, 4d, and lisinopril was evaluated through molecular dynamics simulations using the Desmond module of Schrödinger 2024-3.^22^ These simulations were conducted over a period of 200 ns, with the resulting trajectories meticulously analyzed and interpreted.

**Fig. 18:**
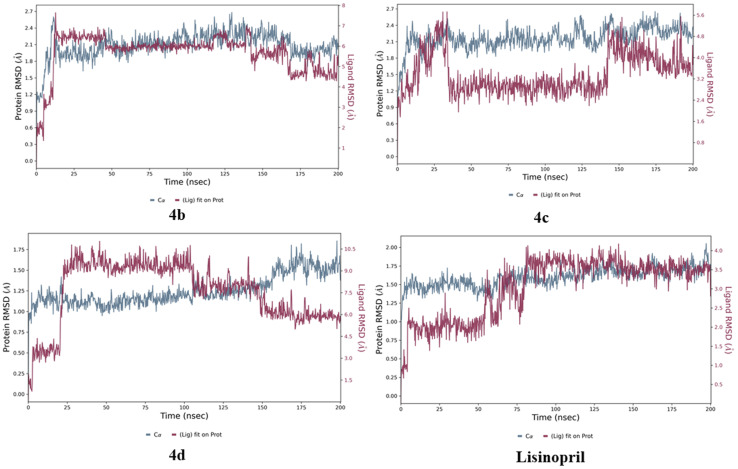
Protein–ligand RMSD plots of compounds 4b, 4c, and 4d and lisinopril.

The protein-ligand RMSD plots in Fig. 18 show that for compound 4b, a brief equilibration phase was observed, marked by slight fluctuations up to 50 ns. The RMSD stabilized between 50 and 150 ns, indicating that the ligand successfully achieved a binding conformation that was well suited to the protein's active site. During the final segment of the simulation (150–200 ns), only minor variations were noted, with the RMSD consistently remaining within the range of 1–3 Å. These findings underscore the potential of compound 4b as a leading candidate for further optimization.

In the case of compound 4c, the system displayed slight fluctuations between 0 and 30 ns, indicating an initial equilibration phase. The RMSD exhibited variability from 150 to 170 ns, indicating a period of moderate stability characterized by minimal oscillations. However, complete equilibration was not attained during the final phase of the simulation.

In the case of compound 4d, minor fluctuations were noted up to 25 ns, indicating an initial equilibration period. However, between 25 and 100 ns, significant differences emerged in the RMSD of both the ligand and the protein, suggesting that the ligand had shifted from its original binding position. Notably, the system appeared to stabilize at 100 ns, with the protein–ligand RMSD remaining constant until approximately 150 ns. After this point, fluctuations resumed, which may indicate either temporary binding interactions or ongoing conformational changes. These observations suggest that compound 4d may exhibit less stable binding characteristics.

For lisinopril, the early stabilization phase was characterized by slight changes in both the ligand and protein RMSD up to 50 ns. The system tended to reach equilibrium at 125 ns, with only minor deviations observed for the remainder of the simulation period. These findings indicate that lisinopril continues to bind to the target protein in a stable manner, reinforcing its role as a proven inhibitor. In contrast, compound 4b demonstrated rapid equilibration, achieving stability by 50 ns and maintaining that stability until 150 ns. The minor variations observed toward the end of the simulation indicate a consistent binding conformation for compound 4b. In comparison, lisinopril exhibited greater fluctuations, with RMSD deviations persisting until approximately 70 ns. Following this, the equilibration process commenced at approximately 125 ns and continued until the simulation's conclusion was reached. When comparing compound 4b with lisinopril, which requires a longer time to stabilize, it is clear that compound 4b exhibited faster equilibration and more robust stability. The minimal changes noted for compound 4b toward the end of the simulation further support the notion of a well-preserved binding conformation. The ligand–protein RMSD plots are depicted in Fig. 18.

**Fig. 19:**
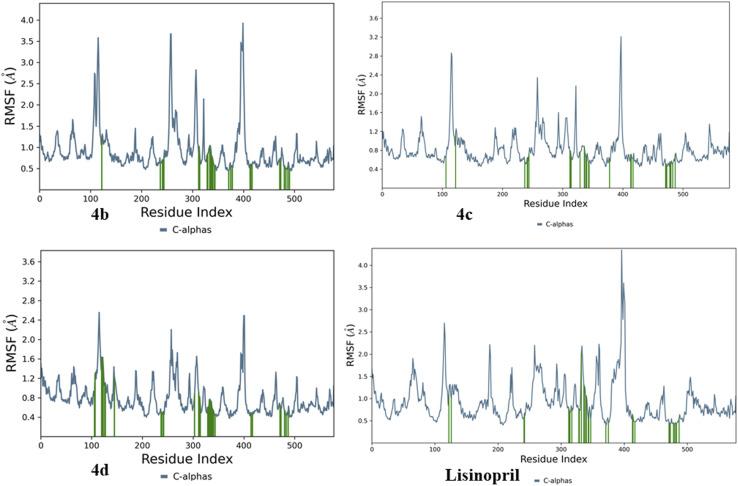
Protein RMSFs of compounds 4b, 4c, and 4d and lisinopril.

To evaluate the flexibility and mobility of various amino acid residues in the presence of compounds 4b, 4c, and 4d and the standard lisinopril, we concentrated on the protein root mean square fluctuation (RMSF), as shown in Fig. 19. The findings indicate that a higher RMSF value is associated with increased flexibility, whereas a lower RMSF value signifies greater rigidity in the amino acid residues, and a green dash highlights the flexibility and mobility of amino acid residues in contact with compounds.

The protein RMSF plot reveals that compound 4b has higher RMSF values of 3.6, 3.8, and 4.0 Å, indicating enhanced flexibility among amino acid residues 120, 250, and 400, respectively. Conversely, an RMSF value below 0.5 Å implies that the amino acid residues exhibit greater rigidity. Similarly, amino acid residues affected by compound 4c presented increased protein RMSF values of 2.8 and 3.2 Å for residues 110 and 400, with the lowest protein RMSF observed at 0.4 Å. Compound 4d presented elevated protein RMSF values of 2.3 and 2.5 Å, alongside a minimal RMSF of 0.4 Å. The standard lisinopril exhibited greater flexibility in the amino acid residues, as evidenced by the higher protein RMSF values of 2.6, 2.4, and 4.5 Å, with the least flexibility occurring below 0.5 Å.

In summary, the results clearly indicate that amino acid residues are more flexible and mobile in compound 4b and lisinopril. The protein RMSF plots of compounds 4b, 4c, and 4d and lisinopril are depicted in Fig. 19.

**Fig. 20:**
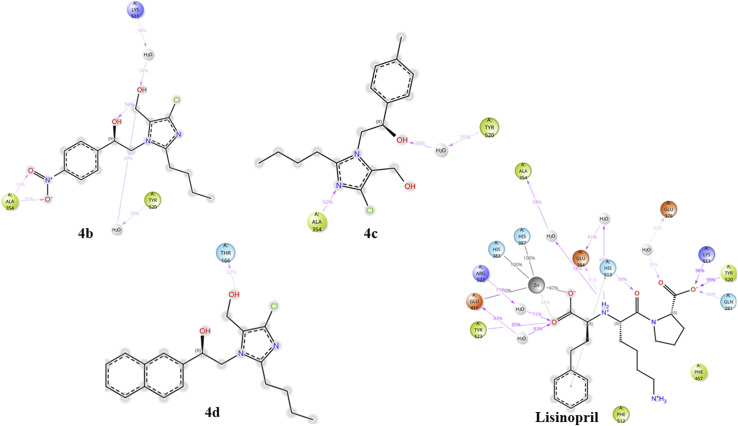
Protein–ligand contacts of 4b, 4c, 4d, and lisinopril.

As shown in Fig. 20, the contacts between proteins and ligands were analyzed. The data revealed that the standard lisinopril presented the greatest number of protein–ligand contacts, outperforming the other compounds. Like lisinopril, compound 4b demonstrated a significant number of contacts, whereas compound 4c exhibited moderate levels of contacts. In contrast, among the compounds evaluated, compound 4d presented the lowest number of protein–ligand contacts.

**Fig. 21:**
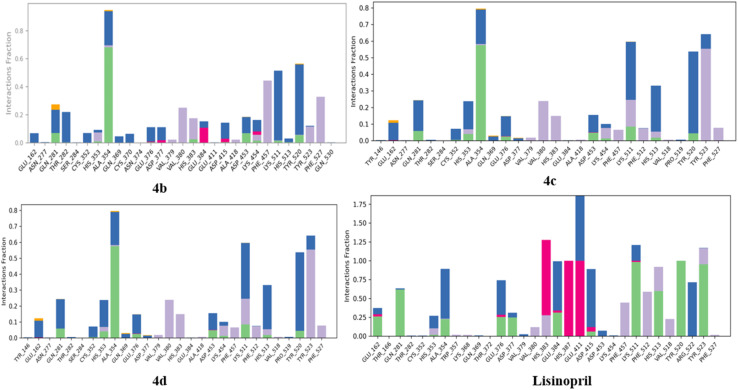
Protein–ligand contact histograms of compounds 4b, 4c, 4d, and lisinopril.

The protein–ligand contact histograms for compounds 4b, 4c, and 4d and lisinopril are presented in Fig. 21. This histogram effectively shows the various types of protein–ligand interactions, including hydrogen bonds (indicated in green), hydrophobic interactions (in gray), ionic interactions (in pink), water bridges (in blue), and halogen bond formations (in yellow). A value of 0.1 on the *y*-axis signifies that a specific interaction occurs for 10% of the simulation time; similarly, 0.2 corresponds to 20%, 0.5 to 50%, and 1.0 to 100%. These findings clearly indicate that lisinopril formed a greater number of interactions, followed by compound 4b, whereas compounds 4c and 4d displayed relatively similar interaction profiles.

#### 2.6.4 Drug likeness prediction

The QikProp module in Schrödinger 2024-3 was used to evaluate the drug likeness of all the derivatives,^23^ and the results are shown in Table 8.

**Table 8:** Drug likeness of compounds 4a–f^a^

Compound	Molecular weight	Donor hydrogen bond	Acceptor hydrogen bond	*Q*p log *P*_o/W_	*n*_violations_	*Q*_P_ log *P*_C16_	*Q*_P_ log *P*_oct_
Rule	<500	≤5	≤10	≤5	≤1	4–18	8–43
4a	338.83	2	5.6	3.533	0	11.135	16.263
4b	353.80	2	5.9	2.79	0	11.64	17.669
4c	322.83	2	4.9	3.775	0	10.854	15.734
4d	358.87	2	4.9	4.354	0	12.623	17.64
4e	343.25	2	4.9	3.818	0	11.033	15.818
4f	326.80	2	4.9	3.57	0	10.052	15.413

a *Q*_p_ log *P*_o/W_: octanol/water partition coefficient, *Q*_P_ log *P*_C16_: hexadecane/gas partition coefficient, *Q*_P_ log *P*_oct_: octanol/gas partition coefficient, *n*_violations_: violations from Lipinski's rule.

### 2.7 Structure–activity relationship study

The structure–activity relationship (SAR) analysis of *N*-substituted 2-butyl-4-chloro-1*H*-imidazole derivatives concerning ACE inhibition demonstrated that electron-withdrawing groups (EWGs) increase activity. The highest level of inhibition was observed for compound 4b, which contains a 4-NO_2_ group. Density functional theory (DFT) simulations support these findings, indicating that 4b strongly interacts with the enzyme, characterized by the smallest energy gap of 3.5247 eV and the highest electrophilicity index of 6.3922. Conversely, electron-donating groups (EDGs), such as 4-CH_3_ (4c) and 4-OCH_3_ (4a), show a reduction in activity, likely because the increased electron density hinders enzyme binding. A bulky hydrophobic naphthalene derivative (4d) displayed moderate activity, suggesting potential steric hindrance and hydrophobic interactions. The least amount of inhibition was recorded for halogen derivatives such as 4-Cl (4e) and 4-F (4f), which can be attributed to their reduced electronic contributions, possibly stemming from their high electronegativity and smaller size. Importantly, molecular dynamics simulations of 4b, encompassing RMSD and RMSF analyses, closely resemble those of lisinopril, indicating minimal conformational variations and stable binding to the enzyme. This evidence underscores the potential of 4b as an ACE inhibitor, implying that it forms a highly stable enzyme–ligand complex.

## 3 Experimental

### 3.1 Materials and methods

All the reagents and chemicals used were obtained from Merck, Loba Chemie, BLD Pharma, and SRL and were used without extra purification unless otherwise stated. The improvement of the reaction was supervised by precoated aluminum sheets using ethyl acetate and toluene as mobile phase solvents (1 : 3) with visualization in a UV cabinet. ^1^H and ^13^C NMR spectra were recorded on a Bruker AM 400 MHz spectrometer, TMS was employed as an internal standard, DMSO-*d*_6_ was used as a solvent for NMR analysis, and a Waters ARC with a 2998 SQ detector 2 was used to collect the ESI + *m*/*z* fragments.

### 3.2 Chemistry

#### 3.2.1 Synthesis of *N*-substituted-2-butyl-4-chloro-1*H*-imidazole-5-carbaldehydes (3a–f)

An equimolar mixture of 2-butyl-4-chloro-1*H*-imidazole-5-carbaldehyde (1) and various phenacyl bromides (2a–f) was stirred at room temperature in the presence of K_2_CO_3_ as a base (1.2 equivalents) and DMF as the solvent to yield intermediates (3a–f). The progress of the reaction was supervised using TLC with ethyl acetate and toluene as the mobile phases. Upon completion of the reaction, water was added, and the mixture was extracted with ethyl acetate. The organic layer was then collected and concentrated under reduced pressure to obtain compounds 3a–f.

#### 3.2.2 Synthesis of *N*-substituted-(2-butyl-4-chloro-1*H*-imidazole)methanol derivatives (4a–f)

Compounds 3a–f were subsequently reduced using sodium borohydride (0.1 mmol) in methanol at room temperature. Afterwards, the reaction mixture was stirred for about thirty minutes, and the solid product was precipitated by adding cold water. The resulting mixture was then filtered, washed with cold water and toluene, and recrystallized from ethyl acetate to obtain the compounds 4a–f.

### 3.3 Spectral analysis

#### 3.3.1 2-(2-Butyl-4-chloro-5-(hydroxymethyl)-1*H*-imidazol-1-yl)-1-(4-methoxyphenyl)ethan-1-ol (4a)

White solid. Yield: 92%. M.P: 187–190 °C. FT-IR (*ν* cm^−1^): 794 (C–Cl), 1597 (C=C), 1675 (C=N), 2833 (aliphatic C–H), 2954 (aromatic C–H), 3181 (OH). ^1^H NMR (400 MHz, DMSO-*d*_6_, ppm): *δ* 0.88 (t, 3H, *J* = 8.0 Hz), *δ* 1.26–1.35 (m, 2H), *δ* 1.47–1.60 (m, 2H), *δ* 2.36–2.47 (m, 2H), *δ* 3.75 (s, 3H), *δ* 3.97–4.05 (m, 2H), *δ* 4.34–4.44 (m, 2H), *δ* 4.83–4.87 (m, H), *δ* 5.18 (t, H, *J* = 4.0 Hz), *δ* 5.70 (d, H, *J* = 4.0 Hz). *δ* 6.92 (d, 2H, *J* = 8.0 Hz), *δ* 7.24 (d, 2H, *J* = 8.0 Hz). ^13^C NMR (100 MHz, DMSO-*d*_6_, ppm): *δ* 14.22, 22.33, 26.15, 29.43, 51.72, 51.88, 55.57, 71.85, 114.06, 125.47, 126.02, 127.57, 135.14, 148.11, 159.21. Molecular formula: [C_17_H_23_ClN_2_O_3_]. ESI mass (*m*/*z*): 339.47 (M + H)^+^.

#### 3.3.2 2-(2-Butyl-4-chloro-5-(hydroxymethyl)-1*H*-imidazol-1-yl)-1-(4-nitrophenyl)ethan-1-ol (4b)

White solid. Yield: 89%. M.P: 179–182 °C. FT-IR (*ν* cm^−1^): 770 (C–Cl), 1344 and 1517 (N–O), 1601 (C=C), 1662 (C=N), 2873 (aliphatic C–H), 2957 (aromatic C–H), 3162 (OH). ^1^H NMR (400 MHz, DMSO-*d*_6_, ppm): *δ* 0.85 (t, 3H, *J* = 8.0 Hz), *δ* 1.23–1.33 (m, 2H), *δ* 1.45–1.59 (m, 2H), *δ* 2.40–2.46 (m, 2H), *δ* 4.03–4.12 (m, 2H), *δ* 4.40–4.49 (m, 2H), *δ* 5.08–5.12 (m, H), *δ* 5.25 (t, H, *J* = 4.0 Hz), *δ* 6.08 (d, H, *J* = 4.0 Hz). *δ* 7.63 (d, 2H, *J* = 8.0 Hz), *δ* 8.23 (d, 2H, *J* = 8.0 Hz). ^13^C NMR (100 MHz, DMSO-*d*_6_, ppm): *δ* 14.21, 22.29, 26.17, 29.42, 51.04, 51.93, 71.60, 123.89, 125.66, 125.96, 127.79, 147.42, 148.28, 150.87. Molecular formula: [C_16_H_20_ClN_3_O_4_]. ESI mass (*m*/*z*): 354.29 (M + H)^+^.

#### 3.3.3 2-(2-Butyl-4-chloro-5-(hydroxymethyl)-1*H*-imidazol-1-yl)-1-(*p*-tolyl)ethan-1-ol (4c)

White solid. Yield: 93%. M.P: 155–158 °C. FT-IR (*ν* cm^−1^): 754 (C–Cl), 1609 (C=C), 1681 (C=N), 2870 (aliphatic C–H), 2956 (aromatic C–H), 3304 (OH). ^1^H NMR (400 MHz, DMSO-*d*_6_, ppm): *δ* 0.85 (t, 3H, *J* = 8.0 Hz), *δ* 1.25–1.34 (m, 2H), *δ* 1.48–1.58 (m, 2H), *δ* 2.29 (s, 3H), *δ* 2.39–2.48 (m, 2H) *δ* 3.97–4.06 (m, 2H), *δ* 4.33–4.45 (m, 2H), *δ* 4.84–4.88 (m, H), *δ* 5.17 (t, H, *J* = 4.0 Hz), *δ* 5.71 (d, H, *J* = 4.0 Hz), *δ* 7.14–7.22 (m, 4H). ^13^C NMR (100 MHz, DMSO-*d*_6_, ppm): *δ* 14.22, 21.19, 22.32, 26.14, 29.42, 51.68, 51.88, 72.10, 125.46, 126.01, 126.31, 129.19, 137.08, 140.18, 148.14. Molecular formula: [C_17_H_23_ClN_2_O_2_]. ESI mass (*m*/*z*): 323.27 (M + H)^+^.

#### 3.3.4 2-(2-Butyl-4-chloro-5-(hydroxymethyl)-1*H*-imidazol-1-yl)-1-(naphthalen-1-yl)ethan-1-ol (4d)

Buff white solid. Yield: 85%. M.P: 171–174 °C. FT-IR (*ν* cm^−1^): 747 (C–Cl), 1584 (C=C), 1634 (C=N), 2930 (aliphatic C–H), 3050 (aromatic C–H), 3328 (OH). ^1^H NMR (400 MHz, DMSO-*d*_6_, ppm): *δ* 0.82 (t, 3H, *J* = 8.0 Hz), *δ* 1.20–1.29 (m, 2H), *δ* 1.41–1.57 (m, 2H), *δ* 2.41–2.47 (m, 2H), *δ* 4.10–4.20 (m, 2H), *δ* 4.40–4.51 (m, 2H), *δ* 5.08–5.12 (m, H), *δ* 5.24 (t, H, *J* = 4.0 Hz), *δ* 5.94 (d, H, *J* = 4.0 Hz). *δ* 7.49–7.55 (m, 3H), *δ* 7.87–7.93 (m, 4H). ^13^C NMR (100 MHz, DMSO-*d*_6_, ppm): *δ* 14.18, 22.29, 26.22, 29.42, 51.54, 51.95, 72.42, 124.80, 124.95, 125.56, 126.03, 126.36, 126.70, 128.04, 128.26, 128.27, 133.02, 133.24, 140.74, 148.22. Molecular formula: [C_20_H_23_ClN_2_O_2_]. ESI mass (*m*/*z*): 359.47 (M + H)^+^.

#### 3.3.5 2-(2-Butyl-4-chloro-5-(hydroxymethyl)-1*H*-imidazol-1-yl)-1-(4-chlorophenyl)ethan-1-ol (4e)

White solid. Yield: 82%. M.P: 186–189 °C. FT-IR (*ν* cm^−1^): 770 (C–Cl), 1582 (C=C), 1662 (C=N), 2867 (aliphatic C–H), 2956 (aromatic C–H), 3268 (OH). ^1^H NMR (400 MHz, DMSO-*d*_6_, ppm): *δ* 0.87 (t, 3H, *J* = 8.0 Hz), *δ* 1.24–1.34 (m, 2H), *δ* 1.45–1.59 (m, 2H), *δ* 2.35–2.48 (m, 2H), *δ* 4.0–4.08 (m, 2H), *δ* 4.36–4.46 (m, 2H), *δ* 4.92–4.96 (m, H), *δ* 5.21 (t, H, *J* = 4.0 Hz), *δ* 5.87 (d, H, *J* = 4.0 Hz). *δ* 7.35 (d, 2H, *J* = 8.0 Hz), *δ* 7.42 (d, 2H, *J* = 8.0 Hz). ^13^C NMR (100 MHz, DMSO-*d*_6_, ppm): *δ* 14.22, 22.32, 26.14, 29.41, 51.37, 51.89, 71.55, 125.57, 125.98, 128.32, 128.65, 132.52, 142.13, 148.19. Molecular formula: [C_16_H_20_Cl_2_N_2_O_2_]. ESI mass (*m*/*z*): 343.26 (M + H)^+^.

#### 3.3.6 2-(2-Butyl-4-chloro-5-(hydroxymethyl)-1*H*-imidazol-1-yl)-1-(4-fluorophenyl)ethan-1-ol (4f)

White solid. Yield: 80%. M.P: 148–151 °C. FT-IR (*ν* cm^−1^): 774 (C–Cl), 1214 (C–F), 1505 (C=C), 1600 (C=N), 2864 (aliphatic C–H), 2963 (aromatic C–H), 3272 (OH). ^1^H NMR (400 MHz, DMSO-*d*_6_, ppm): *δ* 0.88 (t, 3H, *J* = 8.0 Hz), *δ* 1.26–1.35 (m, 2H), *δ* 1.49–1.60 (m, 2H), *δ* 2.41–2.49 (m, 2H), *δ* 3.99–4.09 (m, 2H), *δ* 4.35–4.46 (m, 2H), *δ* 4.91–4.96 (m, H), *δ* 5.21 (t, H, *J* = 4.0 Hz), *δ* 5.84 (d, H, *J* = 4.0 Hz). *δ* 7.17–7.22 (m, 2H), *δ* 7.35–7.38 (m, 2H). ^13^C NMR (100 MHz, DMSO-*d*_6_, ppm): *δ* 14.22, 22.31, 26.14, 29.43, 51.53, 51.88, 71.57, 115.33, 125.54, 125.99, 128.34, 139.34, 148.17, 160.85, 163.26. Molecular formula: [C_16_H_20_ClFN_2_O_2_]. ESI mass (*m*/*z*): 327.29 (M + H)^+^.

### 3.4 Biology

#### 3.4.1 Preparation of sheep kidney acetone powder

Sheep kidney acetone powder was prepared in the laboratory according to a modified protocol by Hector *et al.*^24^ Fresh sheep kidney tissue was homogenized with prechilled acetone (1 : 3) and vacuum-filtered using a Buchner funnel. Re-extraction was performed until the solid residue turned colorless. This residue was air-dried and sifted to obtain a uniform powder, which was stored in an airtight container at 0–4 °C.

#### 3.4.2 Extraction of sheep ACE

ACE was extracted from the prepared acetone powder as described by Jimsheena *et al.*^25^ One gram of acetone powder was extracted *via* 10 mL of sodium borate buffer (0.05 M, pH 8.2) containing 0.3 M sodium chloride and 0.5% Triton X-100 at 4 °C for 18 hours. This extract was later centrifuged at 15 000*g* for 60 minutes at 4 °C. The supernatant was collected and dialyzed against the same buffer without Triton X-100 (3 L, the buffer was changed 3 times) for 24 hours. This extract was stored at −20 °C until further use. The specific activity was 0.8234 units per mg of protein.

#### 3.4.3 Protein estimation

Protein estimation of the enzyme extract was determined by Bradford's method.^26^

#### 3.4.4 Colorimetric assay for ACE activity

ACE activity was calculated by measuring the release of hippuric acid (HA) from the substrate hippuryl-histidyl-leucine (HHL). One unit of ACE activity is defined as the amount of enzyme that releases 1 μmol of HA per minute at 37 °C and pH 8.2. The ACE enzyme extract was preincubated with different concentrations of the test compounds (250 nM to 25 μM) and a standard drug solution (lisinopril, 100–500 nM) for 10 minutes at 37 °C. The reaction was initiated by the addition of sodium borate buffer (0.05 M, pH 8.2) containing 0.3 M sodium chloride and 5 mM HHL. The mixture was incubated at 37 °C for 30 minutes, followed by the addition of 60 μL of 1 M HCl to stop the reaction. This was followed by the addition of 120 μL of pyridine and 60 μL of benzyl sulfonyl chloride (BSC) for color development. The mixture was mixed gently and cooled on ice for 5 minutes. The yellow color developed was measured spectrophotometrically at 410 nm.^25^ The reduced HA concentration in the inhibition reaction compared with the control reaction is expressed as % inhibition, which was calculated as:% inhibition = 100 − (*A*_test_/*A*_control_) × 100,where *A*_test_ = absorbance of the test reaction and *A*_control_ = absorbance of the control reaction.

### 3.5 Crystallography

#### 3.5.1 Single-crystal X-ray diffraction studies

A single crystal of compound 4c was grown from a solvent mixture comprising water (soluble phase) and ethyl acetate (insoluble phase). Single-crystal X-ray diffraction (SCXRD) data for the compounds were collected using an XtaLAB Pro II AFC12 (RINC) kappa diffractometer equipped with Mo-Kα radiation (*λ* = 0.71073 Å). The complete data collection and reduction were performed using CrysAlis Pro. The molecular structure was solved using the intrinsic phasing method implemented in SHELXT and refined by the full-matrix least-squares method on F^2^ using SHELXL using Olex2 software.^27^

Nonhydrogen atoms were identified on the basis of electron density and refined anisotropically to enhance structural accuracy. The hydrogen atoms were positioned geometrically on their respective parent atoms and refined using a riding model to ensure optimal placement.^28^ The geometrical parameters of the crystal structure were calculated using PLATON,^29^ and packing diagrams were generated using MERCURY 4.2.0.^30^ The crystal structure data and refinement parameters are summarized in Table 2.

#### 3.5.2 Hirshfeld surface and interaction energy calculation

Hirshfeld surface analyses were performed using Crystal Explorer 17.5,^31^ to gain insight into the interactions governing the crystal packing. The *d*_norm_ surface was analyzed on the basis of the parameters *d*_e_ and *d*_i_ distances to the nearest atom outside the surface and inside the surface, respectively, providing quantitative information on each contact.^32^ Red and blue spots on the *d*_norm_ surface specify shorter and longer interatomic contacts, respectively.^16^

In addition, 2D fingerprint plots derived from the *d*_norm_ surface help to evaluate individual molecular contacts quantitatively. The enrichment ratio (*E*_XX_) values derived from the Hirshfeld surface quantify the propensity of atomic contacts in crystal structures. The propensity of each contact was explored by calculating the enrichment ratio on the basis of the obtained actual contacts and derived random contacts.^33,34^

Pairwise intermolecular interaction energies were calculated for fragments within a 3.8 Å radius around the central independent fragment. The energy frameworks were constructed using these interaction energy values, allowing visualization of the 3D topology of the molecular structure.^35–37^

#### 3.5.3 Cambridge structural database (CSD) analysis

CSD provides a robust platform for analyzing the conformational behavior of molecules and their structural analogs by offering access to a vast collection of experimentally determined crystal structures. A search of the CSD revealed several derivatives based on the (2-butyl-4-chloro-1*H*-imidazol-5-yl)methanol scaffold. From these, 14 structurally relevant three-dimensional (3D) crystal structures were selected for detailed comparative analysis. To assess the conformational similarity, all 14 structures were superimposed onto compound 4c using molecular overlay techniques. This allowed for a direct comparison of key geometric parameters, including bond angles, bond lengths, and torsional angles.

### 3.6 Computational studies

#### 3.6.1 Molecular docking

The molecular docking study was conducted using the Glide module of Schrodinger 2024-3. The compounds were sketched with a 2D sketcher and prepared in LigPrep. The protein was sourced from the Protein Data Bank and prepared for docking. The ligands and protein were then docked, with a focus on docking scores and interactions with amino acid residues.^13,20^

#### 3.6.2 Molecular dynamics

The Desmond module of the Schrödinger suite 2024-3 was used for molecular dynamics simulations to stabilize the docked complexes of 4b, 4c, and 4d and lisinopril. A SPC model was created to form an orthorhombic box around the complex, and the simulation lasted 200 ns, with the results analyzed *via* simulation interaction diagrams.^13,22^

#### 3.6.3 DFT calculations

Geometry optimization calculations were carried out for compounds *via* DFT using Gaussian 16 software.^38^ The calculation was performed *via* the APFD functional with the 6-311+G (d,p) basis set within the framework of DFT. This study provides insights into the electronic structure, stability, and reactivity of molecular systems. The investigation of the FMOs (HOMO and LUMO), MEP, and key global reactivity descriptors offers valuable information on the electronic distribution and chemical reactivity potential of the molecule, making it relevant for applications in materials science and drug design. The results were visualized using Gauss View 6 software. The energy gap and related reactive parameters were calculated with the help of Koopman's approximation.

## 4 Conclusion

*N*-Substituted-2-butyl-4-chloro-1*H*-imidazole derivatives were successfully designed and synthesized, and the resulting compounds were confirmed *via* various spectral techniques, including ^1^H NMR, ^13^C NMR, FT-IR, and mass spectrometry. Among these compounds (4a–f), compound 4b (the 4-NO_2_ derivative) exhibited the highest *in vitro* ACE inhibitory activity, highlighting the role of electron-withdrawing groups in enhancing bioactivity. Additionally, compound 4c (the 4-CH_3_ derivative) underwent single-crystal X-ray crystallography, Hirshfeld surface, and CSD analysis, which provided detailed information on its molecular structure and packing. All synthesized compounds were further analyzed through molecular docking, molecular dynamics simulations, MEP, and DFT calculations to better elucidate the structure–activity relationships. These computational analyses indicated that compound 4b possessed the highest electrophilicity, the smallest energy gap, and the greatest stability for enzyme binding, aligning well with known ACE inhibitors such as lisinopril. The combined experimental and theoretical outcomes suggest that compound 4b holds significant promise as an ACE inhibitor, with future modifications incorporating additional electron-withdrawing substituents likely to further enhance its bioactivity.

## Ethical statement

All animal procedures were performed in accordance with the Guidelines for Care and Use of Laboratory Animals of “Kasturba Medical College, MAHE, Manipal” and approved by the Institutional Animal Ethics Committee (IAEC) of Kasturba Medical College, Manipal, MAHE (IAEC/KMC/90/2025).

## Author contributions

Manjunath R.: design, conceptualization, synthesis, experiments, data curation, analysis, manuscript original draft. Ashwini Rao: ACE assay and data analysis, Mahesha: single crystal data analysis, DFT studies, and writing. Udaya Kumar A. H: single crystal data collection and analysis, DFT studies, and writing. Sudarshan Acharya: crystallization, CSD collection, analysis, and writing. Padmanabha Udupa E. G.: ACE assay data analysis. Abdul Ajees Abdul Salam: crystallization, CSD analysis, and writing. Sushruta S Hakkimane: resources, data curation, evaluation. Shashikala B. S.: data curation, analysis. Lokanath N. K.: single crystal data evaluation. Santosh L. Gaonkar: supervision, resources, review, and editing.

## Conflicts of interest

There are no known competing interests.

## Supplementary Material

RA-015-D5RA04675K-s001

RA-015-D5RA04675K-s002

## Data Availability

The crystal structure of 4c has been deposited in the Cambridge Crystallographic Data Centre (CCDC) under deposition number 2448889 and is accessible at http://www.ccdc.cam.ac.uk. CCDC 2448889 contains the supplementary crystallographic data for this paper.^39^ The data underlying this study are available in the published article and its SI. See DOI: https://doi.org/10.1039/d5ra04675k.

## References

[cit1] Liu P., Lan X., Yaseen M., Wu S., Feng X., Zhou L., Sun J., Liao A., Liao D., Sun L. (2019). Purification, characterization and evaluation of inhibitory mechanism of ACE inhibitory peptides from pearl oyster (Pinctada fucata martensii) meat protein hydrolysate. Mar. Drugs.

[cit2] Danaei G., Lu Y., Singh G. M., Carnahan E., Stevens G. A., Cowan M. J., Farzadfar F., Lin J. K., Finucane M. M., Rao M. (2014). Cardiovascular disease, chronic kidney disease, and diabetes mortality burden of cardiometabolic risk factors from 1980 to 2010: a comparative risk assessment. Lancet Diabetes Endocrinol..

[cit3] Wang R., Lu X., Sun Q., Gao J., Ma L., Huang J. (2020). Novel ACE inhibitory peptides derived from simulated gastrointestinal digestion *in vitro* of sesame (Sesamum indicum L.) protein and molecular docking study. Int. J. Mol. Sci..

[cit4] Sun S., Xu X., Sun X., Zhang X., Chen X., Xu N. (2019). Preparation and identification of ACE inhibitory peptides from the marine macroalga Ulva intestinalis. Mar. Drugs.

[cit5] Ibrahim H. R., Ahmed A. S., Miyata T. (2017). Novel angiotensin-converting enzyme inhibitory peptides from caseins and whey proteins of goat milk. J. Adv. Res..

[cit6] Alderman C. P. (1996). Adverse effects of the angiotensin-converting enzyme inhibitors. Ann. Pharmacother..

[cit7] Kostis W. J., Shetty M., Chowdhury Y. S., Kostis J. B. (2018). ACE inhibitor-induced angioedema: a review. Curr. Hypertens. Rep..

[cit8] Ustaoğlu G., Erdal E., Karaş Z. (2021). Influence of different anti-hypertensive drugs on gingival overgrowth: A cross-sectional study in a Turkish population. Oral Dis..

[cit9] Tabacova S. (2005). Mode of action: angiotensin-converting enzyme inhibition—developmental effects associated with exposure to ACE inhibitors. Crit. Rev. Toxicol..

[cit10] Baumann M., Baxendale I. R., Ley S. V., Nikbin N. (2011). An overview of the key routes to the best selling 5-membered ring heterocyclic pharmaceuticals. Beilstein J. Org. Chem..

[cit11] Kantevari S., Addla D., Bagul P. K., Sridhar B., Banerjee S. K. (2011). Synthesis and evaluation of novel 2-butyl-4-chloro-1-methylimidazole embedded chalcones and pyrazoles as angiotensin converting enzyme (ACE) inhibitors. Bioorg. Med. Chem..

[cit12] Jallapally A., Addla D., Bagul P., Sridhar B., Banerjee S. K., Kantevari S. (2015). Design, synthesis and evaluation of novel 2-butyl-4-chloroimidazole derived peptidomimetics as Angiotensin Converting Enzyme (ACE) inhibitors. Bioorg. Med. Chem..

[cit13] Manjunath R., Anantharaju P. G., Madhunapantulas S. V., Gaonkar S. L. (2025). Design, synthesis, and biological evaluation of 2-butyl-4-chloroimidazole-derived 1, 3, 4-oxadiazoles: ACE inhibition, anticancer, and antitubercular activities. J. Mol. Struct..

[cit14] Kumar A. U., Pampa K., Kumara K., Hema M., Harohally N. V., Lokanath N. (2022). Structural-property relationship in halogen-bonded Schiff base derivative: Crystal structure, computational and SARS-CoV-2 docking studies. J. Mol. Struct..

[cit15] Bhat D., Spoorthy L., Sharanya R., Siddesh M., Kumar A. U., Lokanath N. (2023). Influence of hydroxyl group in stabilizing the Schiff base crystal structure: Crystal structure, computational and molecular docking studies. J. Mol. Struct..

[cit16] Udaya Kumar A. H., Kumara K., Harohally N. V., Pampa K. J., Lokanath N. K. (2021). Square Planar trans-N2O2 Cu (II) Complex: Synthesis, Crystal Structure, Hirshfeld Surface, DFT, Antimicrobial and Docking Studies. ChemistrySelect.

[cit17] Jeffrey G. A. and Jeffrey G. A., An Introduction to Hydrogen Bonding, Oxford university press, New York, 1997

[cit18] Milani J. F. (2001). The Weak Hydrogen Bond In Structural Chemistry and Biology. J. Am. Chem. Soc..

[cit19] Shaikh S. R., Gawade R. L., Dabke N. B., Dash S. R., Vanka K., Gonnade R. G. (2024). Crystal engineering for intramolecular π–π stacking: Effect of substitution of electron-donating and electron-withdrawing groups on the molecular geometry in conformationally flexible sulfoesters and sulfonamides. CrystEngComm.

[cit20] Yang Y., Yao K., Repasky M. P., Leswing K., Abel R., Shoichet B. K., Jerome S. V. (2021). Efficient exploration of chemical space with docking and deep learning. J. Chem. Theory Comput..

[cit21] Natesh R., Schwager S. L. U., Sturrock E. D., Acharya K. R. (2003). Crystal structure of the human angiotensin-converting enzyme–lisinopril complex. Nature.

[cit22] Bowers K. J. ; Chow E.; Xu H.; Dror R. O.; Eastwood M. P.; Gregersen B. A.; Klepeis J. L.; Kolossvary I.; Moraes M. A. and Sacerdoti F. D., Scalable algorithms for molecular dynamics simulations on commodity clusters. in Proceedings of the 2006 ACM/IEEE Conference on Supercomputing, 2006; p. 84-es

[cit23] Schrödinger . Enhancing drug development with ADME properties prediction. https://www.schrodinger.com/platform/products/qikprop/, accessed 12/09/2024

[cit24] Héctor Luna E. A. (2013). Comparative Study on the N-acylase Activity of Mammalian Kidney Acetone Powders (KAP’s). J. Mex. Chem. Soc.

[cit25] Jimsheena V., Gowda L. R. (2010). Arachin derived peptides as selective angiotensin I-converting enzyme (ACE) inhibitors: structure–activity relationship. Peptides.

[cit26] Bradford M. M. (1976). A rapid and sensitive method for the quantitation of microgram quantities of protein utilizing the principle of protein-dye binding. Anal. Biochem..

[cit27] Dolomanov O. V., Bourhis L. J., Gildea R. J., Howard J. A., Puschmann H. (2009). OLEX2: a complete structure solution, refinement and analysis program. Appl. Crystallogr..

[cit28] Sheldrick G. M. (2008). A short history of SHELX. Found. Crystallogr..

[cit29] Spek A. (2003). Single-crystal structure validation with the program PLATON. Appl. Crystallogr..

[cit30] Macrae C. F., Sovago I., Cottrell S. J., Galek P. T., McCabe P., Pidcock E., Platings M., Shields G. P., Stevens J. S., Towler M. (2020). Mercury 4.0: From visualization to analysis, design and prediction. Appl. Crystallogr..

[cit31] Spackman P. R., Turner M. J., McKinnon J. J., Wolff S. K., Grimwood D. J., Jayatilaka D., Spackman M. A. (2021). CrystalExplorer: a program for Hirshfeld surface analysis, visualization and quantitative analysis of molecular crystals. Appl. Crystallogr..

[cit32] Spackman M. A., Jayatilaka D. (2009). Hirshfeld surface analysis. CrystEngComm.

[cit33] Jelsch C., Ejsmont K., Huder L. (2014). The enrichment ratio of atomic contacts in crystals, an indicator derived from the Hirshfeld surface analysis. IUCrJ.

[cit34] Kumar A. U., Vindya K., Pampa K., Rangappa K., Lokanath N. (2022). Structure-property relationship in thioxotriaza-spiro derivative: Crystal structure and molecular docking analysis against SARS-CoV-2 main protease. J. Mol. Struct..

[cit35] McKinnon J. J., Spackman M. A., Mitchell A. S. (2004). Novel tools for visualizing and exploring intermolecular interactions in molecular crystals. Structural Science.

[cit36] Mackenzie C. F., Spackman P. R., Jayatilaka D., Spackman M. A. (2017). CrystalExplorer model energies and energy frameworks: extension to metal coordination compounds, organic salts, solvates and open-shell systems. IUCrJ.

[cit37] Krishnegowda H. M., Karthik C. S., Kudigana P. J., Mallu P., Neratur L. K. (2020). μ-phenoxide bridged mixed ligand Cu (II) complex: Synthesis, 3D supramolecular architecture, DFT, energy frameworks and antimicrobial studies. Polyhedron.

[cit38] Gaussian 16 Rev. C.01, Wallingford, CT, 2016, https://www.gaussian.com/gaussian16/

[cit39] Manjunath R. Rao A. , Mahesha, Udaya Kumar A. H., Acharya S., Udupa P., Abdul Salam A. A., Hakkimane S. S., Shashikala B. S., Lokanath N. K. and Gaonkar S. L., CCDC 2448889: Experimental Crystal Structure Determination, 2025, 10.5517/ccdc.csd.cc2n68fl.

