# Supplementary material for: Design and synthesis of N-substituted-2-butyl-4-chloro-1H-imidazole derivatives as potential ACE inhibitors: in vitro study, in-silico analysis, and a multifaceted CSD-supported crystallographic investigation
Source: RSC Adv. 2025 Sep 24;15(42):35077–98. doi: 10.1039/d5ra04675k (PMC12459522; doi:10.1039/d5ra04675k)
Supplement: RA-015-D5RA04675K-s001 [file RA-015-D5RA04675K-s001.pdf]

Supplementary Information (SI) for RSC Advances.
This journal is © The Royal Society of Chemistry 2025

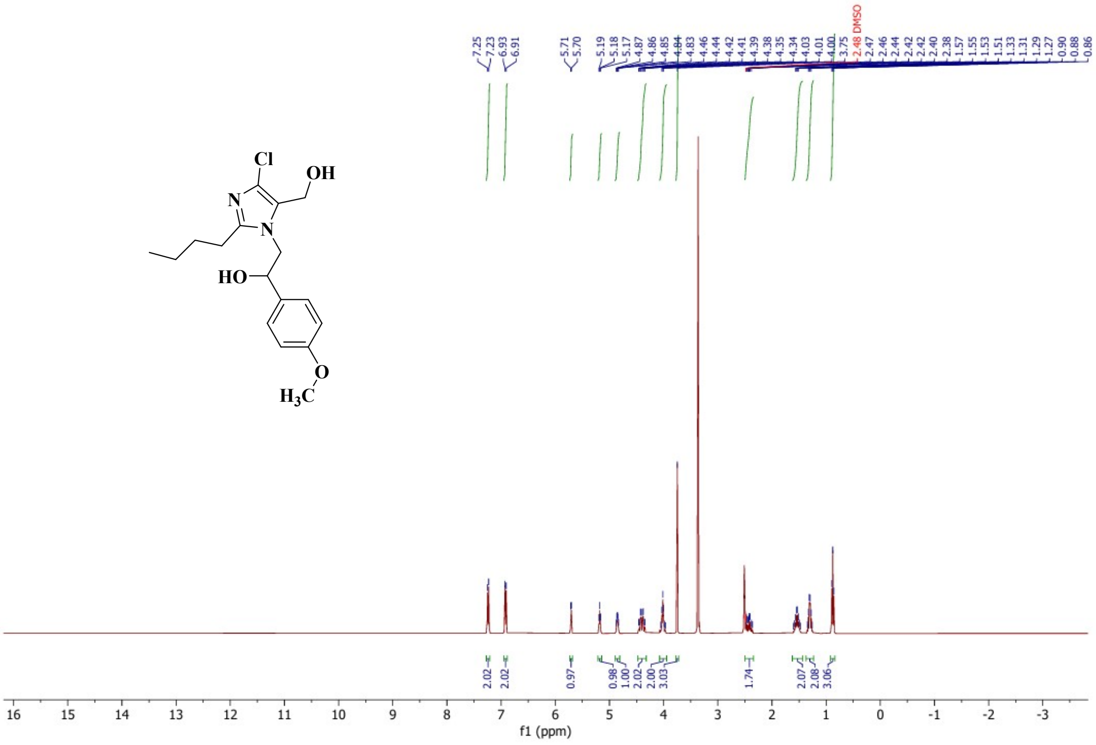

Fig. S1. ^{1}H NMR of **4a**

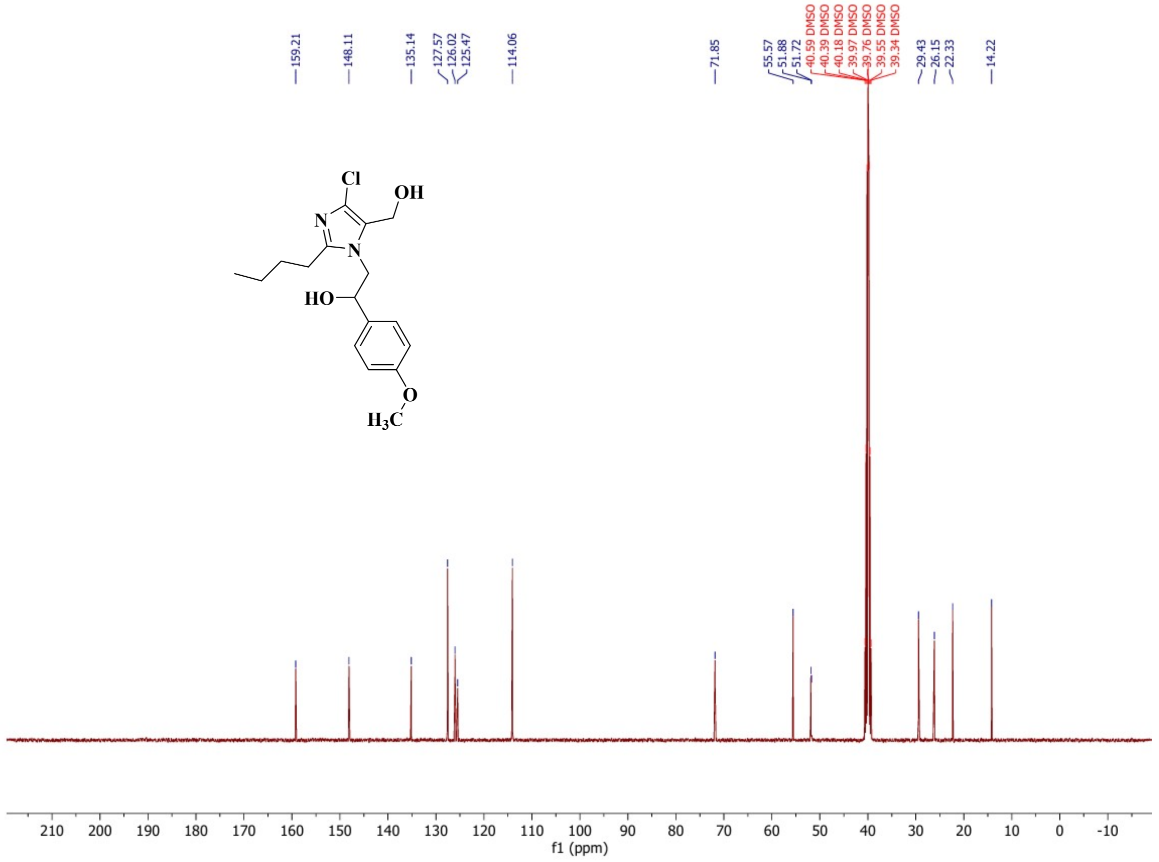

Fig. S2. ^{13}C NMR of **4a**

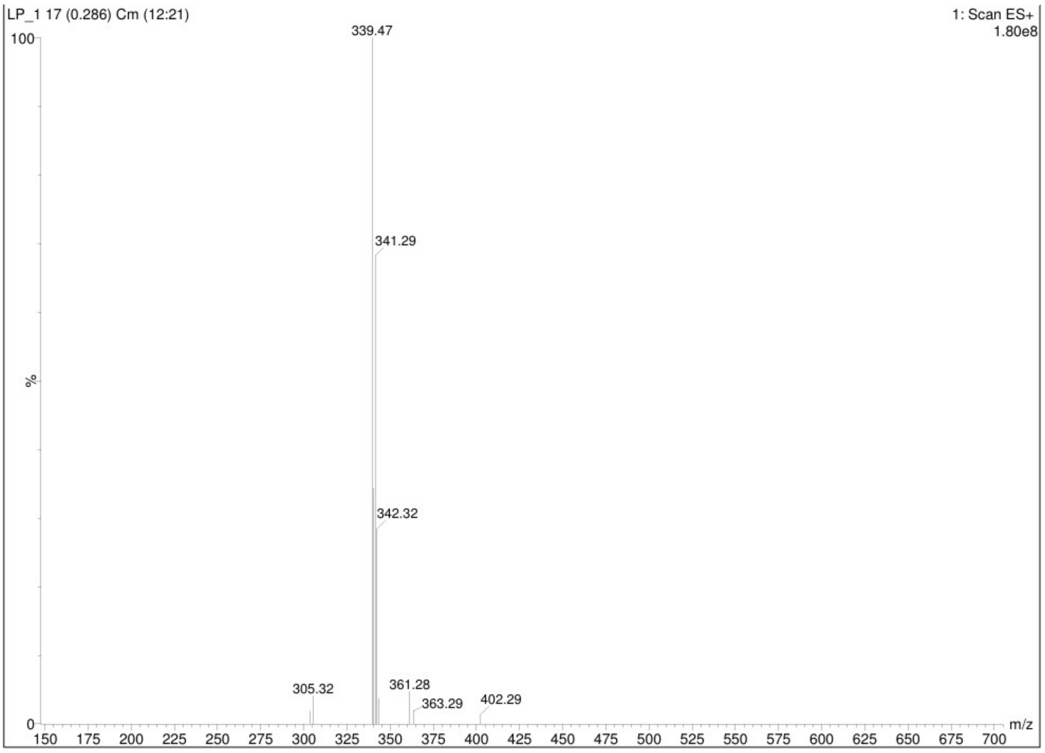

Fig. S3. Mass of **4a**

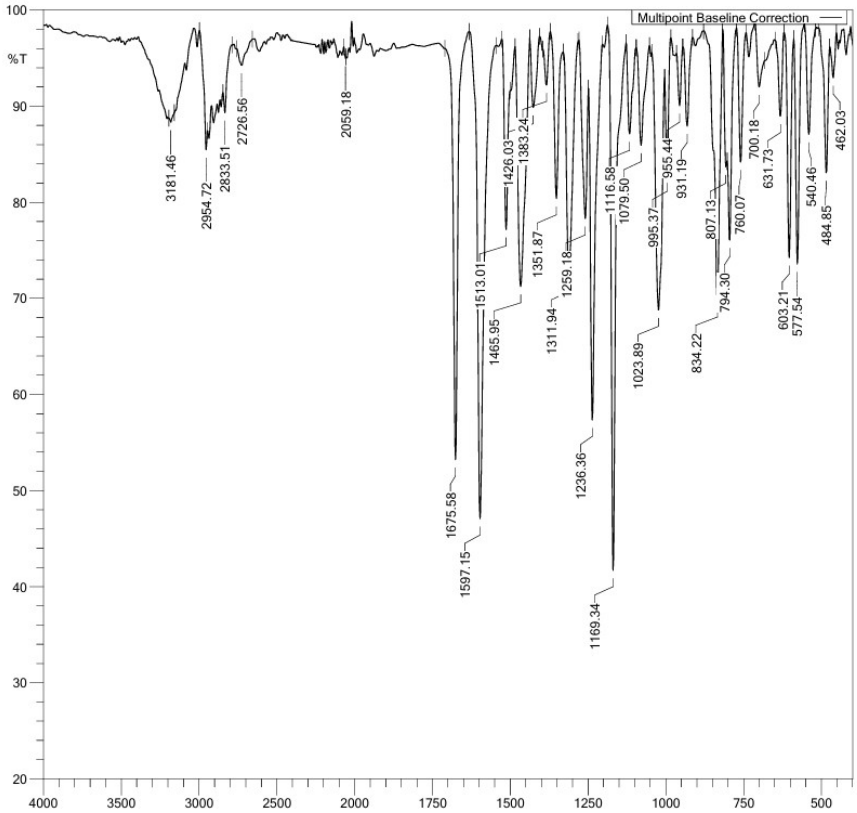

Fig. S4. FTIR of **4a**

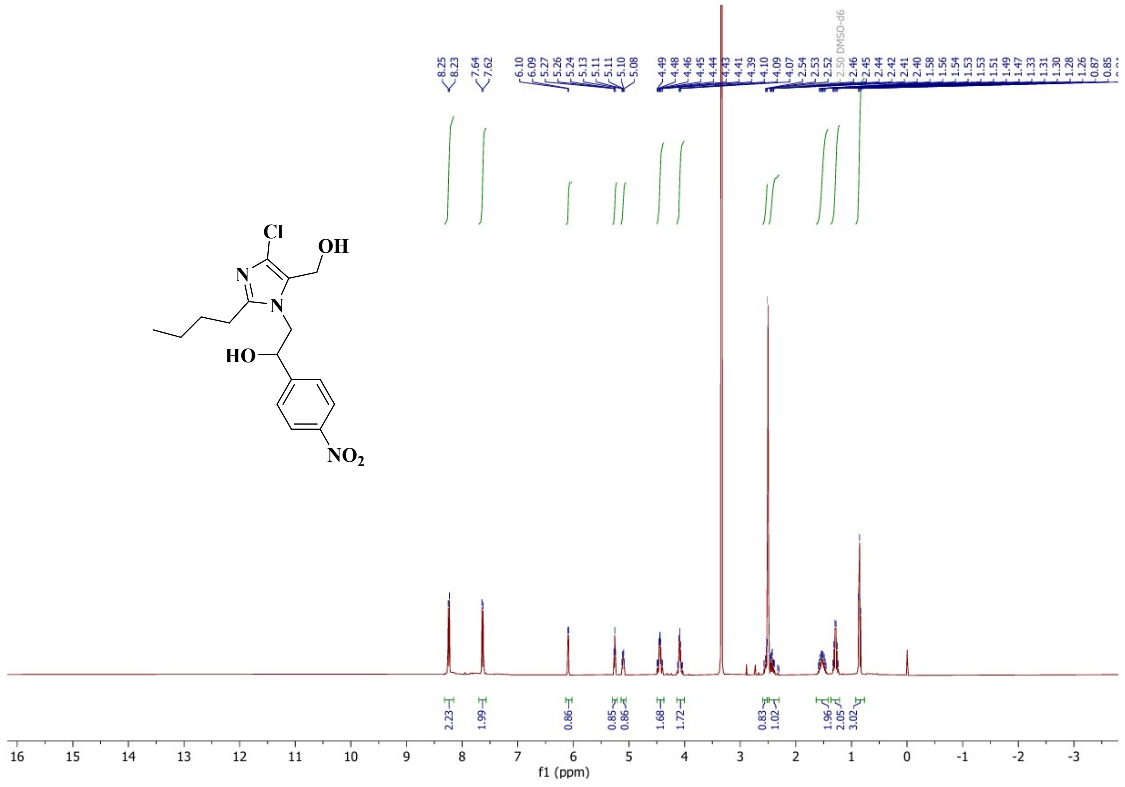

Fig. S5. ^{1}H NMR of **4b**

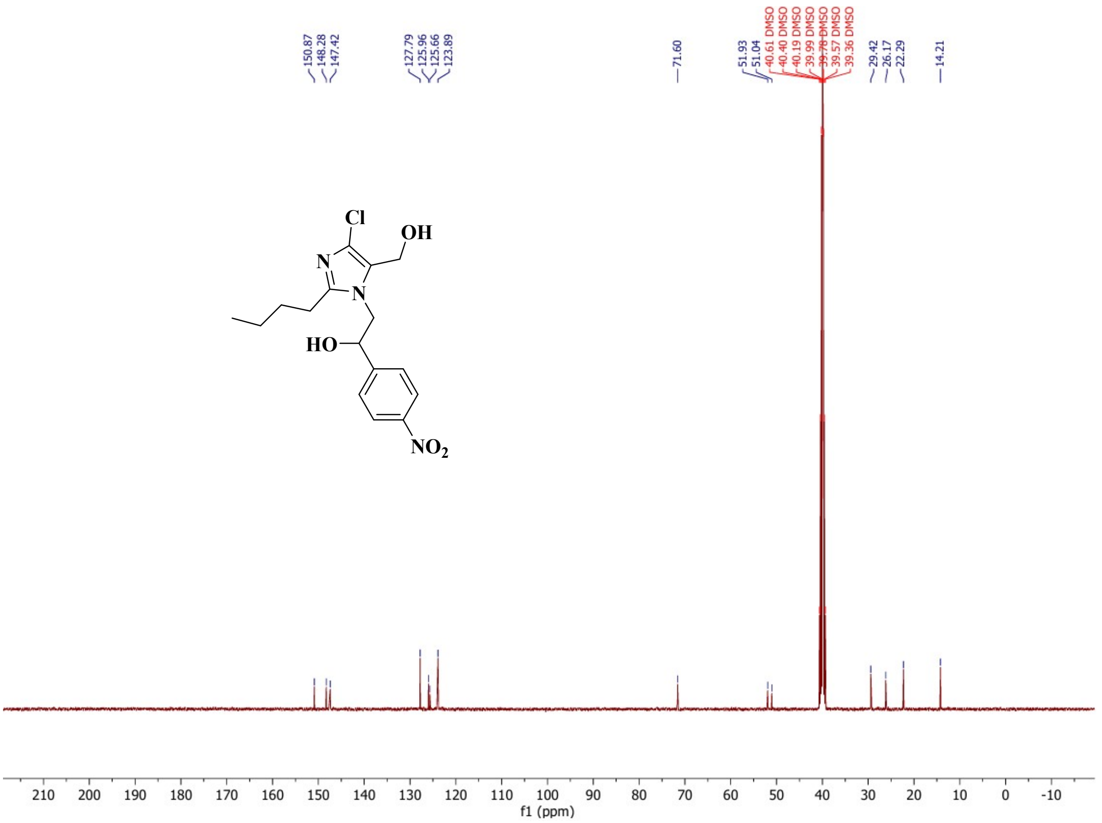

Fig. S6. ^{13}C NMR of **4b**

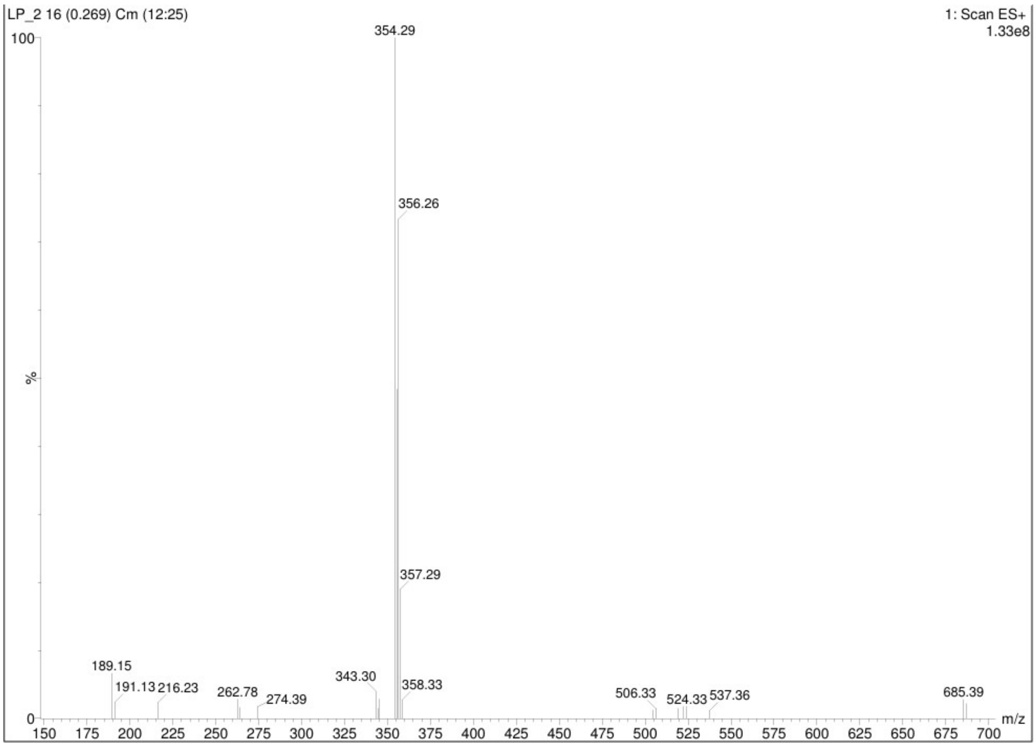

Fig. S7. Mass of **4b**

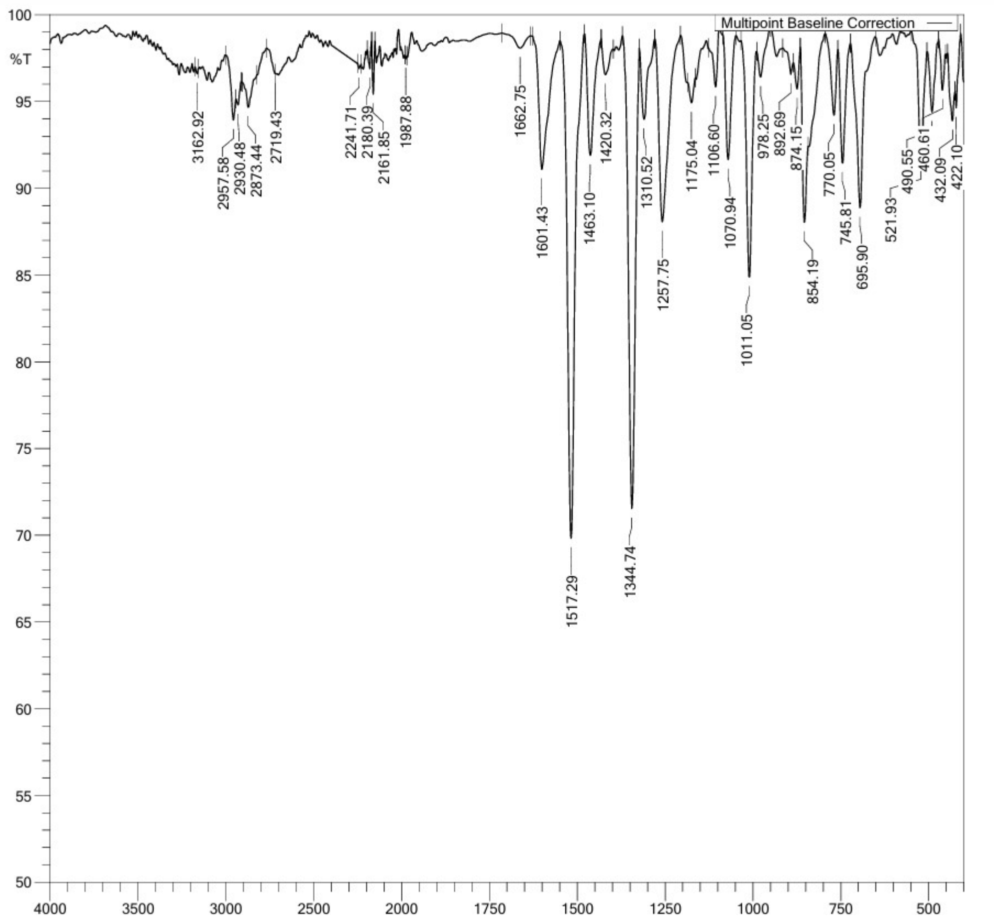

Fig. S8. FTIR of **4b**

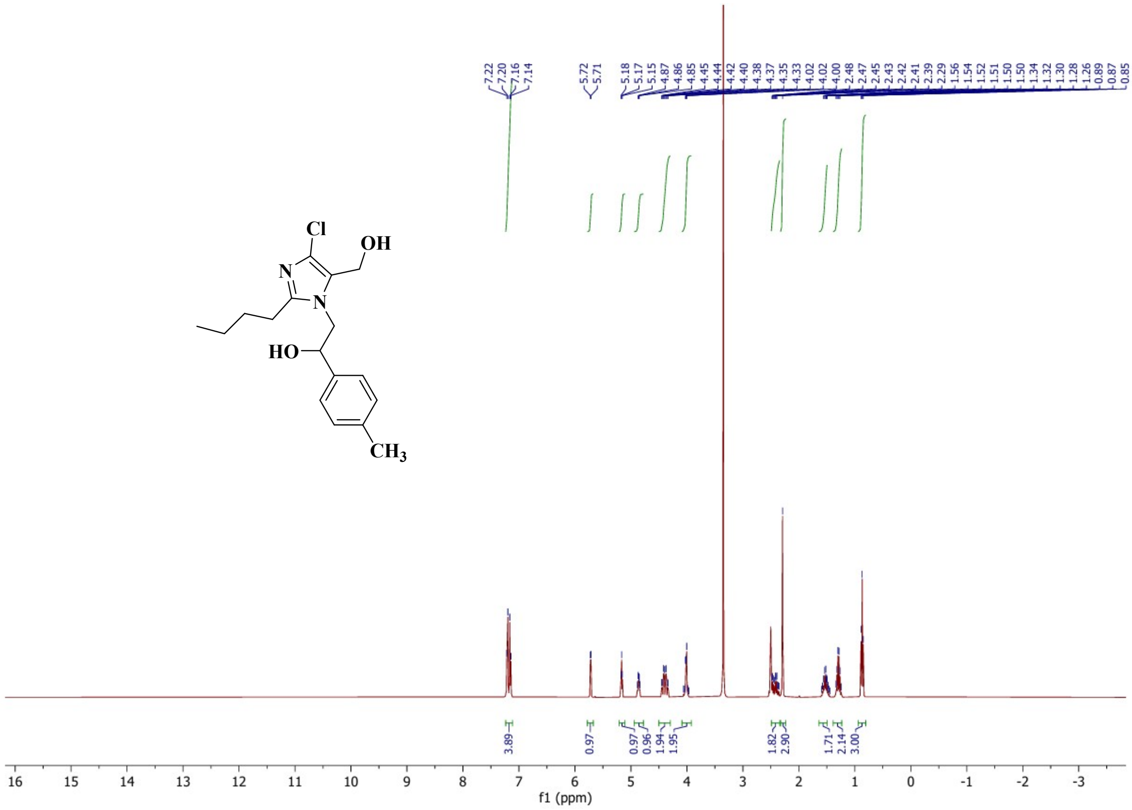

Fig. S9. ^{1}H NMR of **4c**

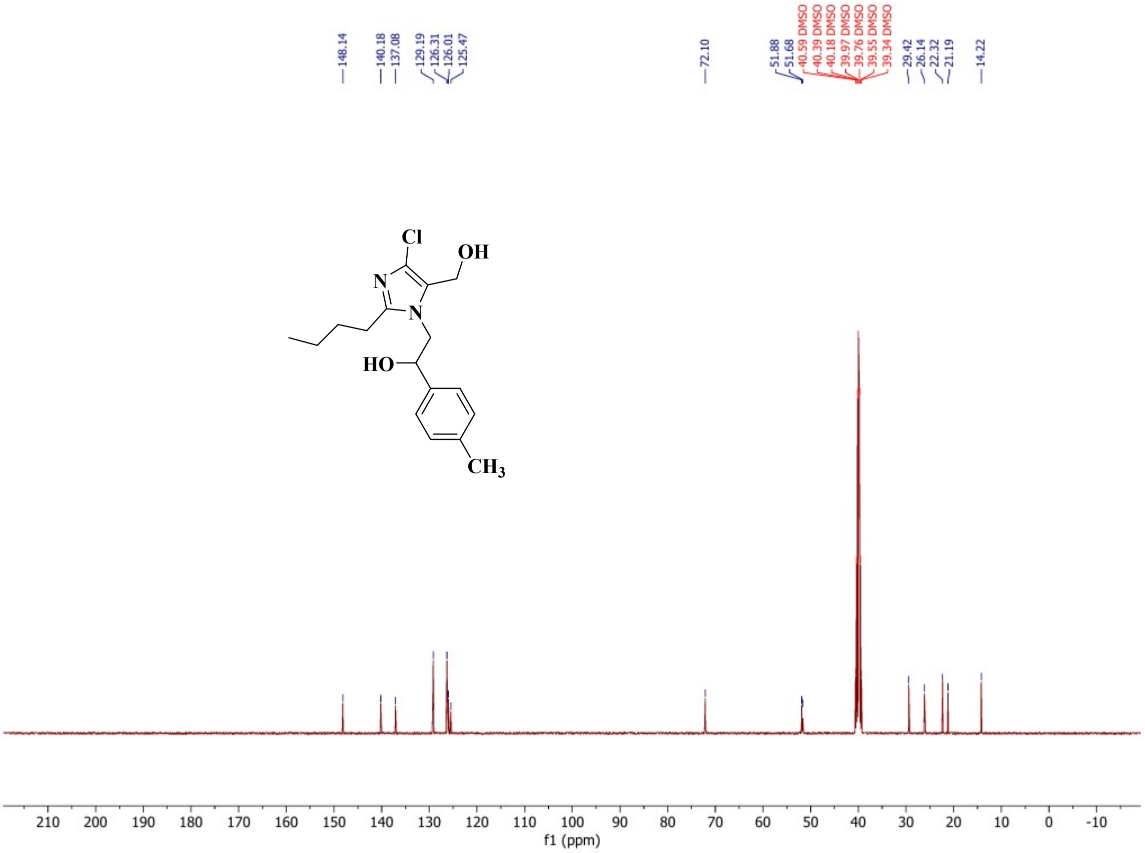

Fig. S10. ^{13}C NMR of **4c**

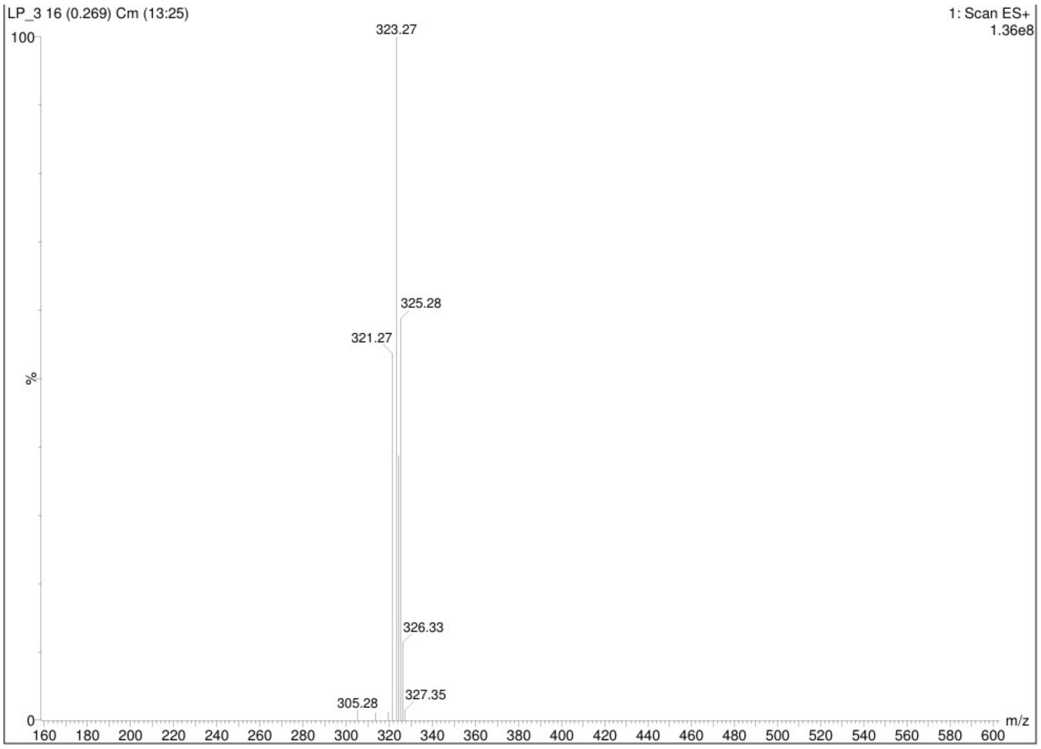

Fig. S11. Mass of **4c**

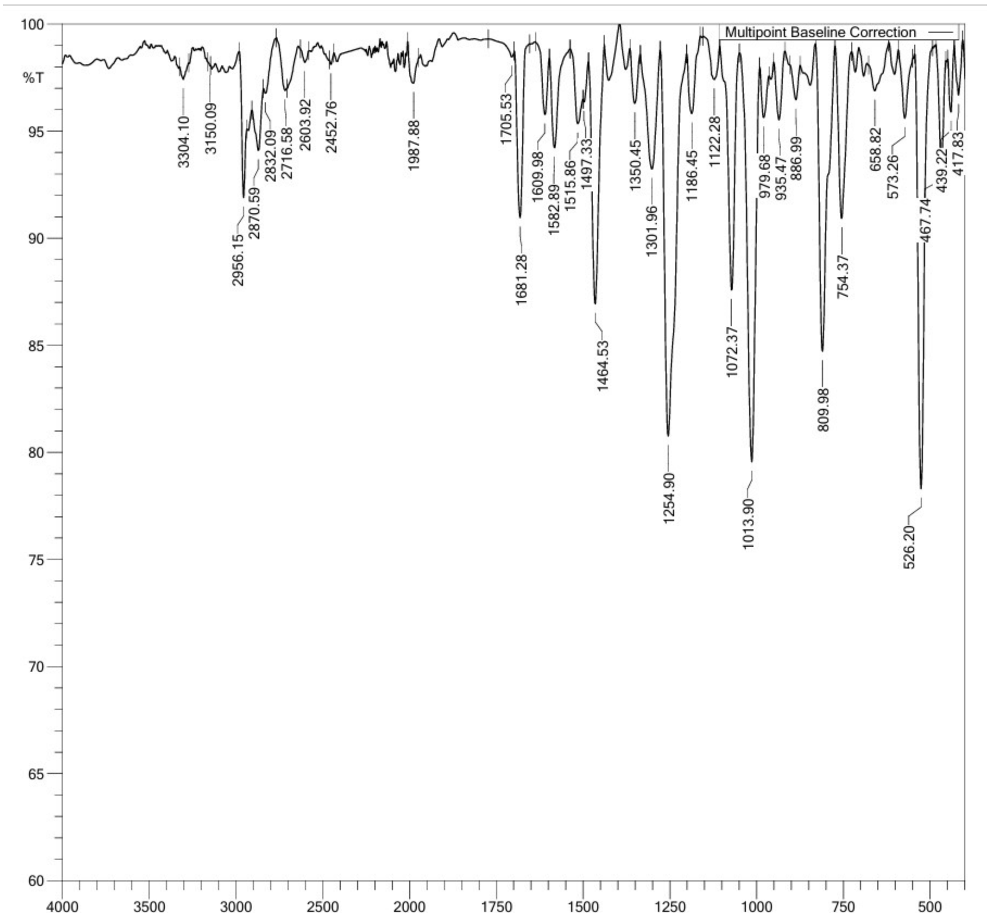

Fig. S12. FTIR of **4c**

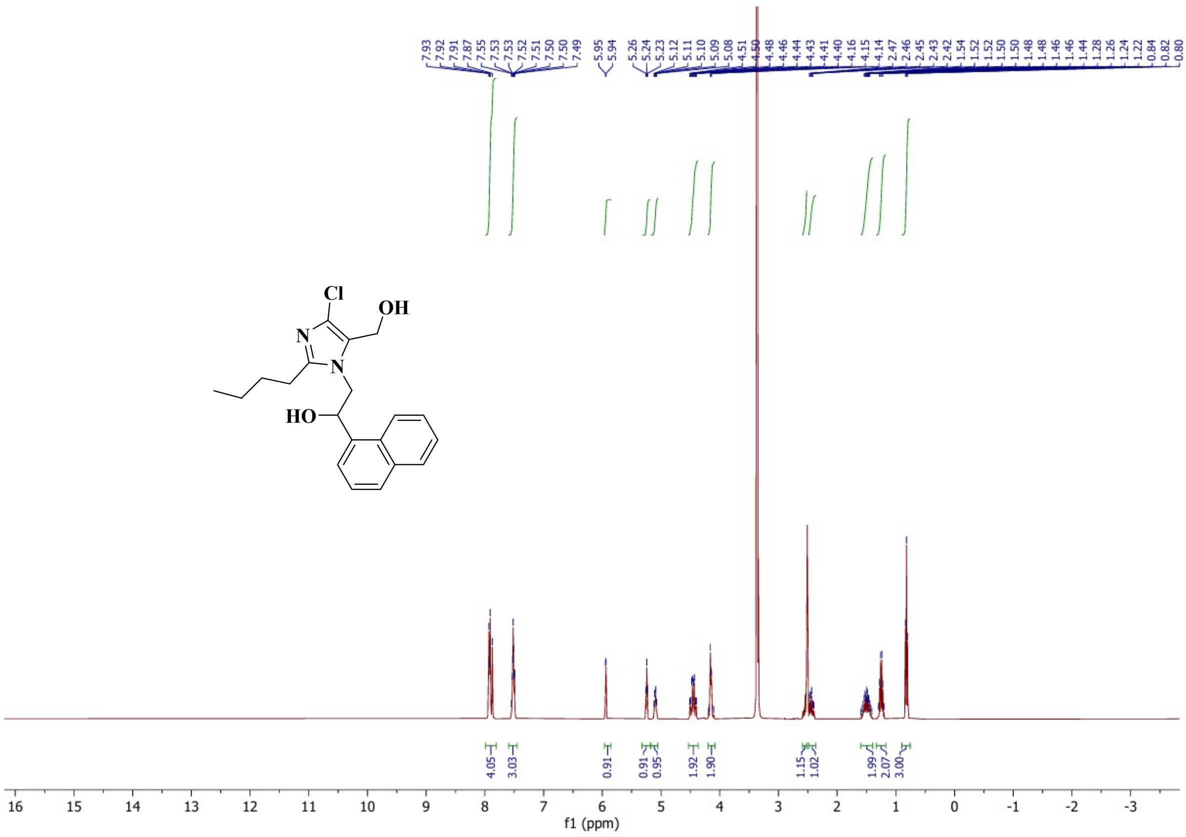

Fig. S13. ^{1}H NMR of **4d**

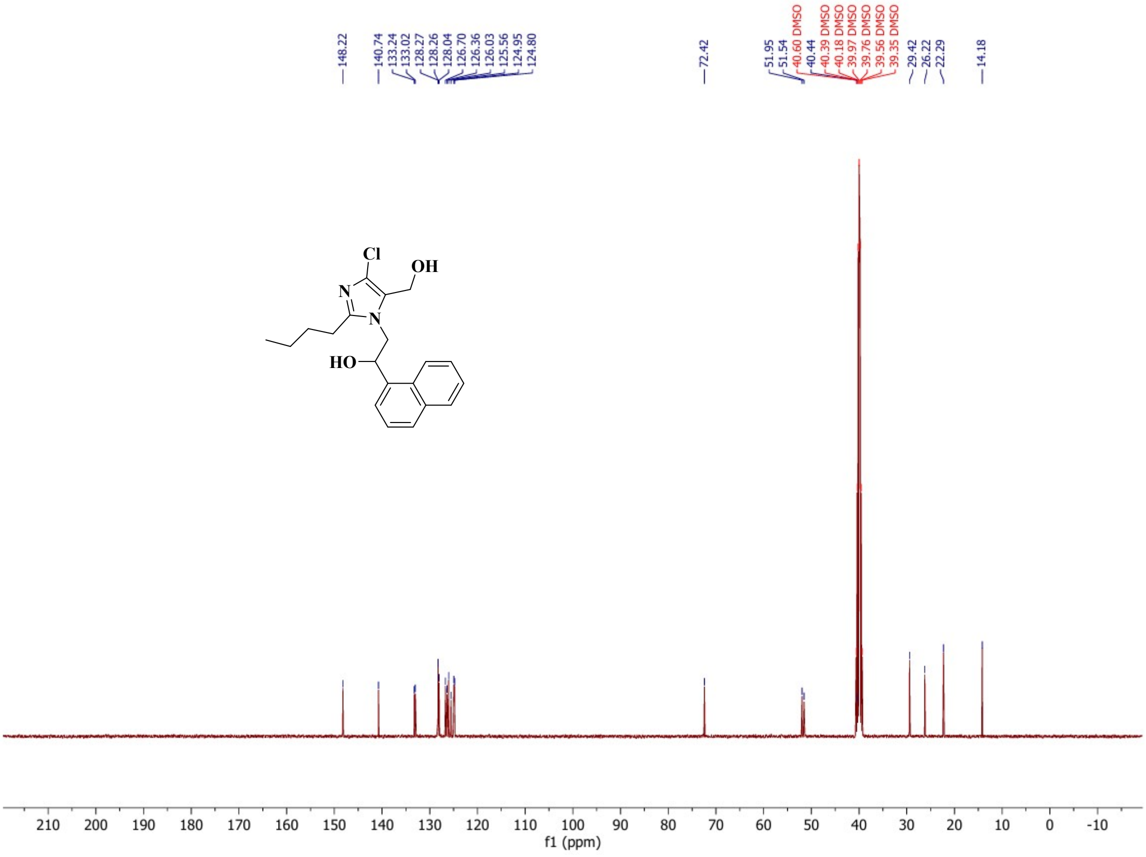

Fig. S14. ^{13}C NMR of **4d**

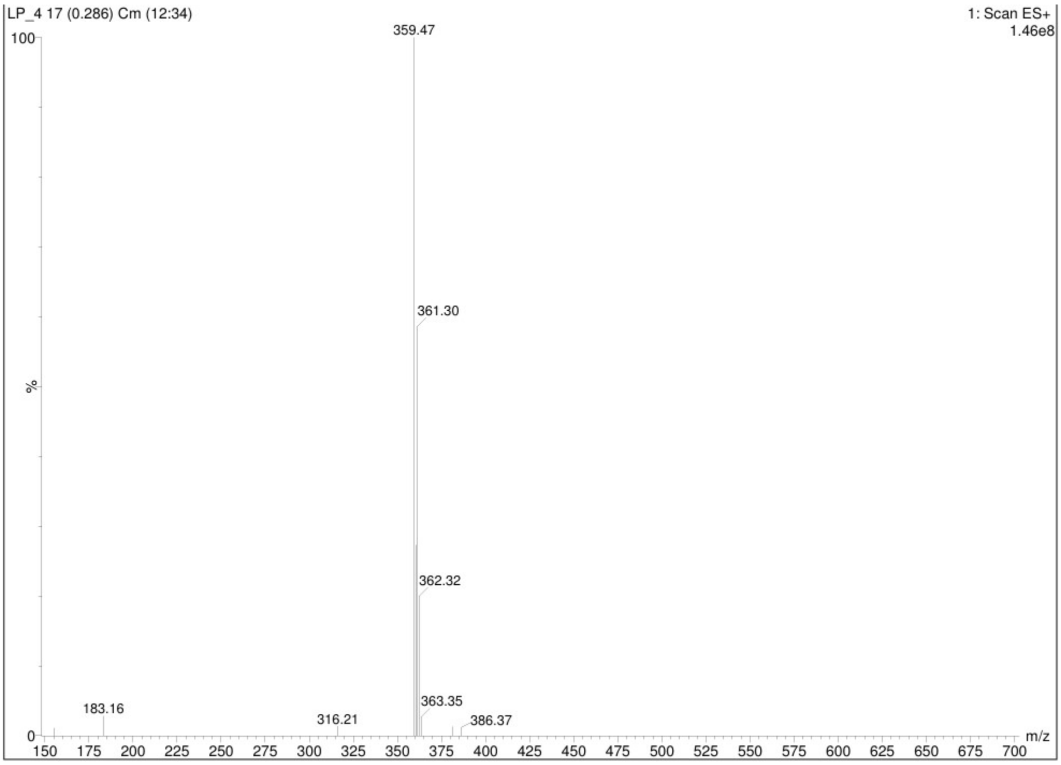

Fig. S15. Mass of **4d**

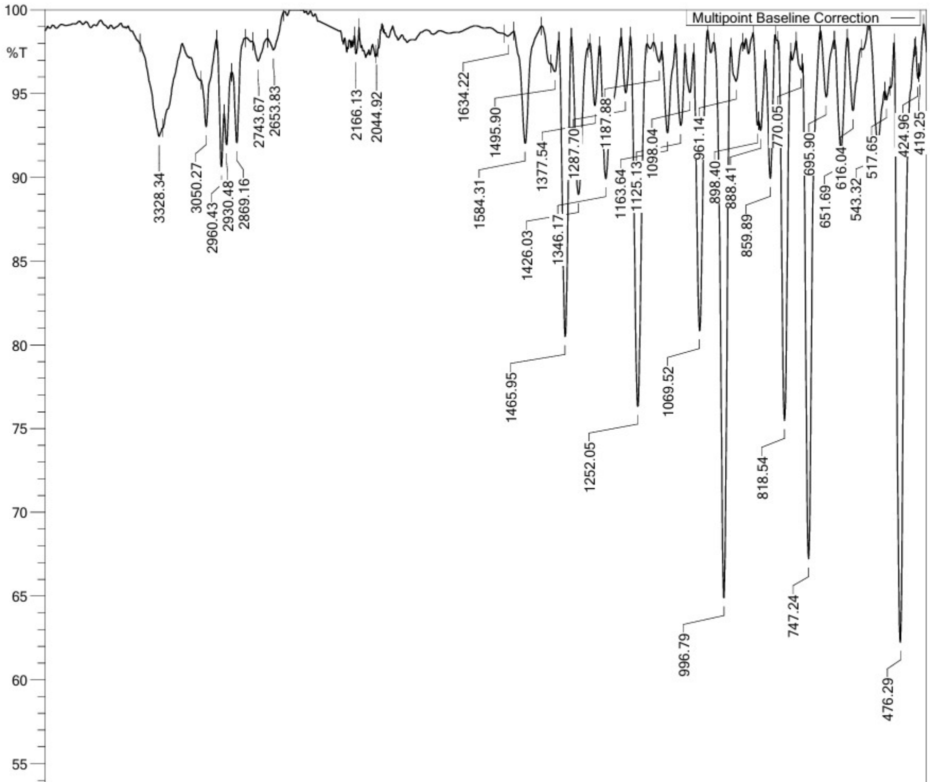

Fig. S16. FTIR of **4d**

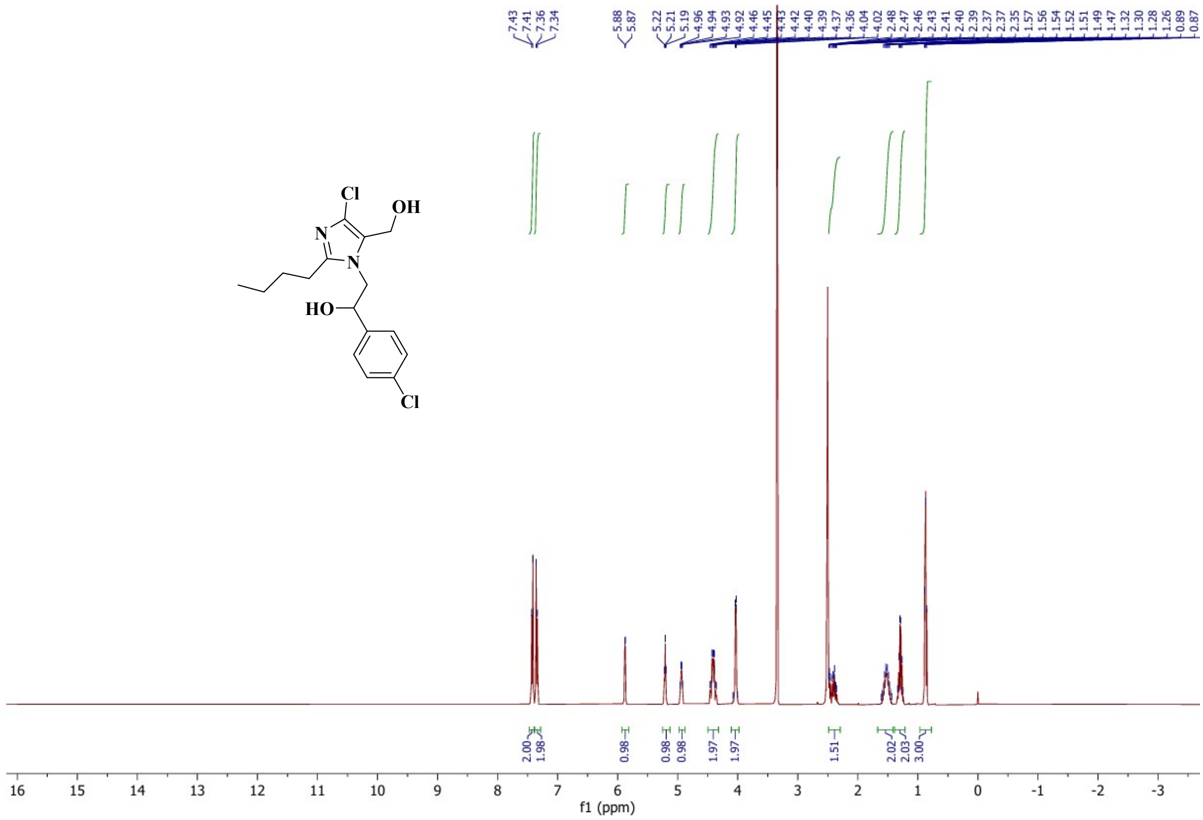

Fig. S17. ^{1}H NMR of **4e**

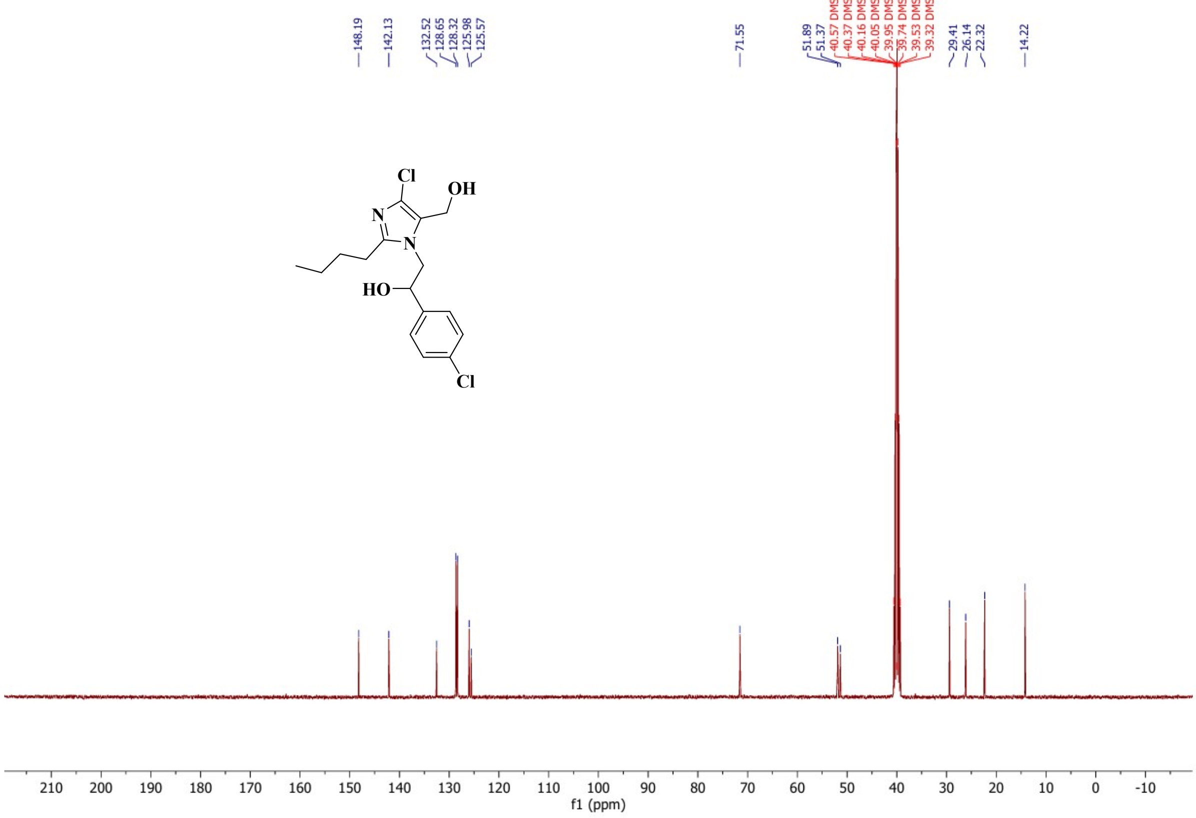

Fig. S18. ^{13}C NMR of **4e**

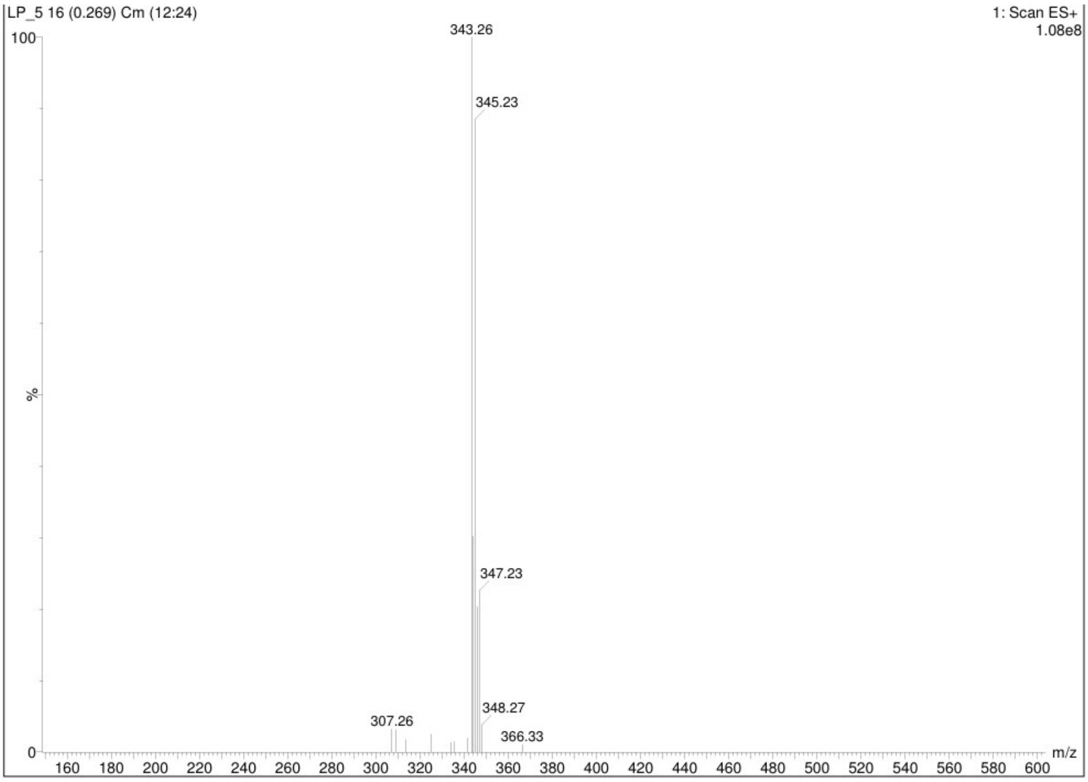

Fig. S19. Mass of **4e**

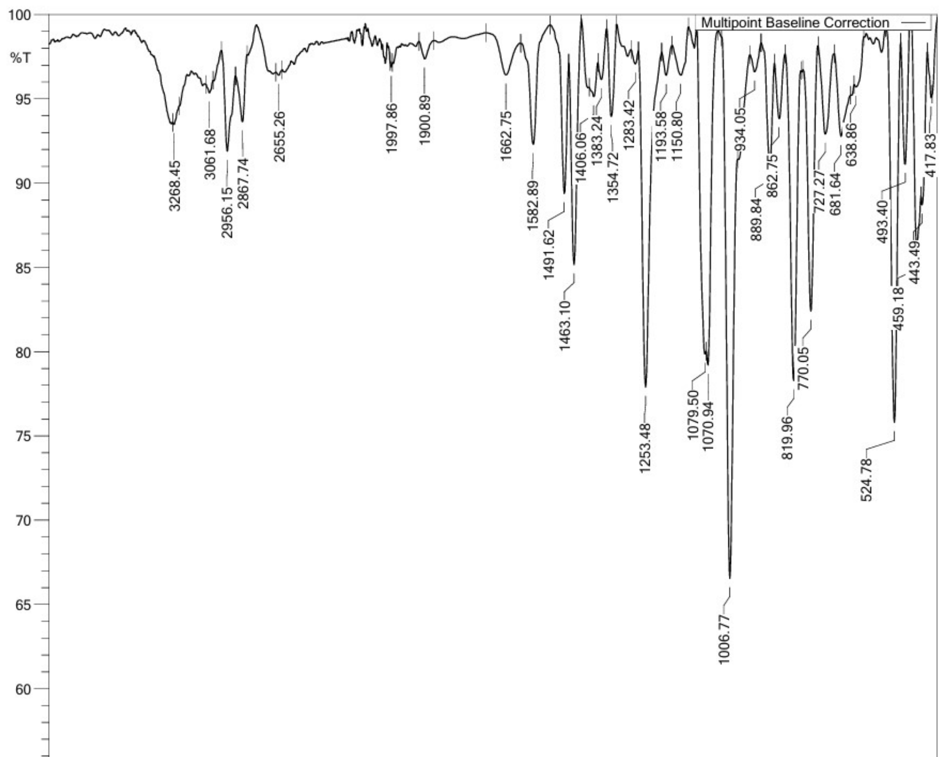

Fig. S20. FTIR of **4e**

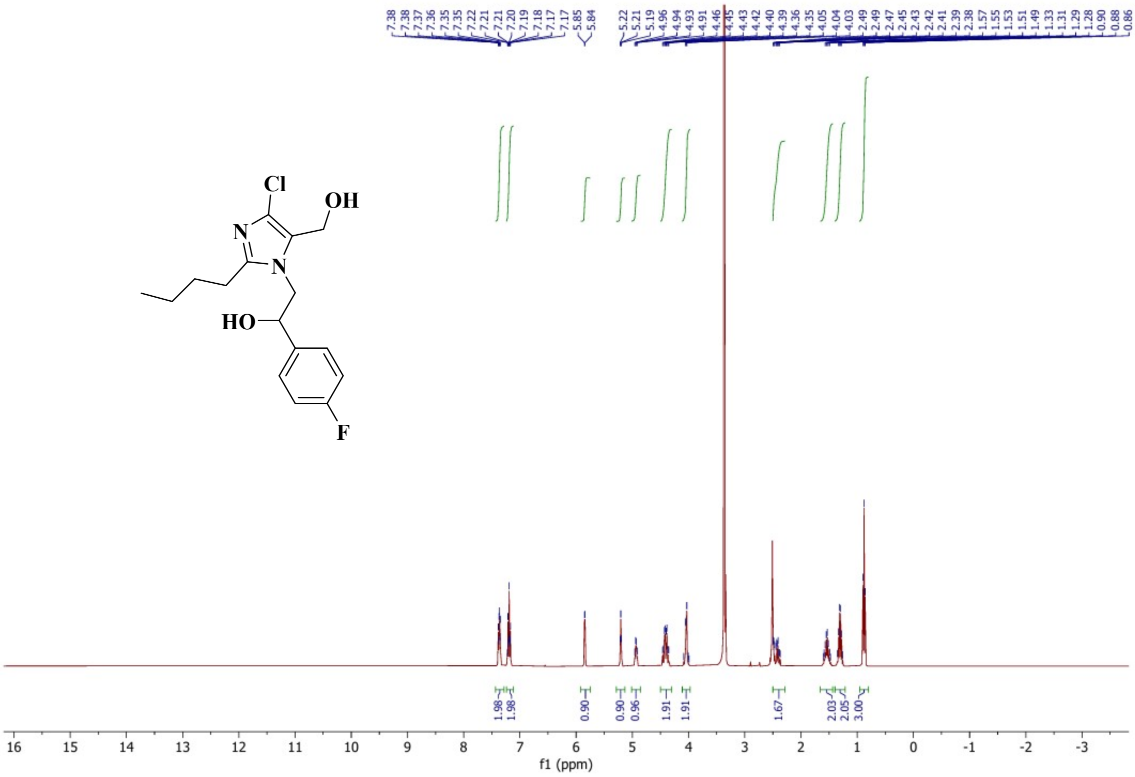

Fig. S21. ^{1}H NMR of **4f**

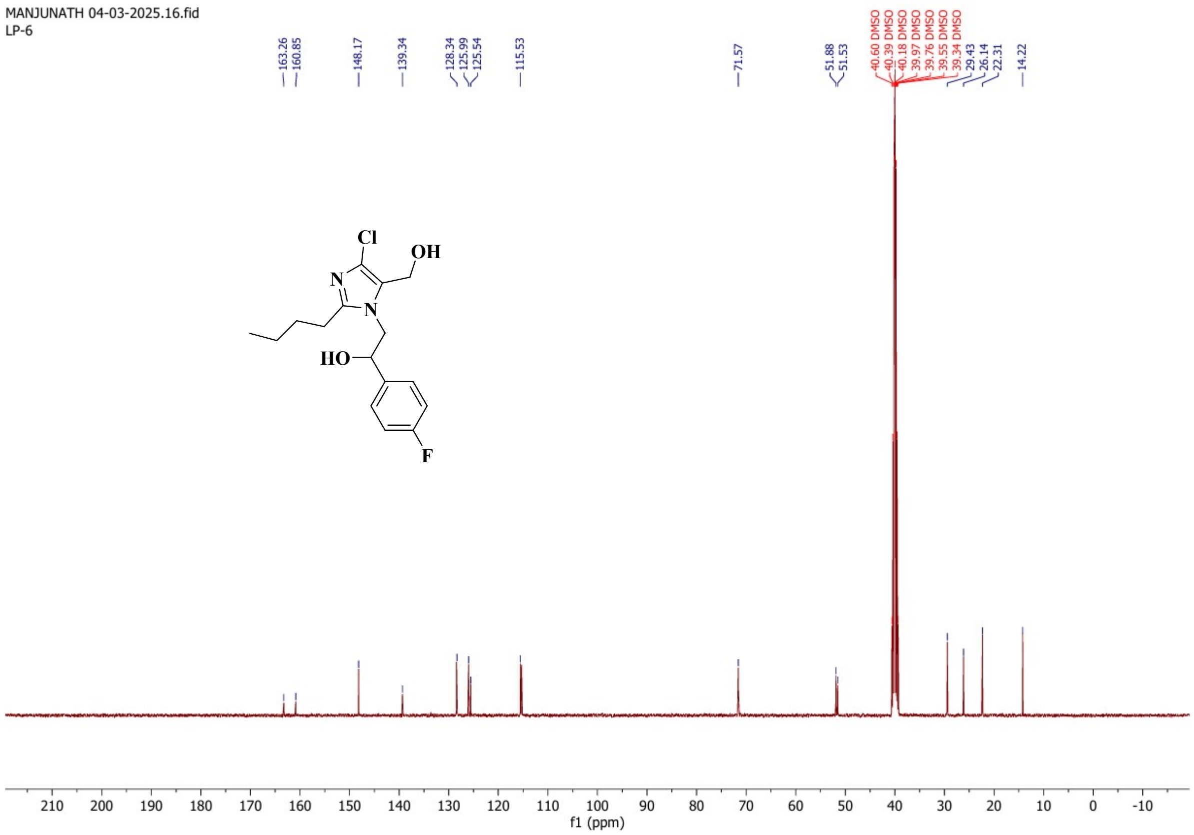

Fig. S22. ^{13}C NMR of **4f**

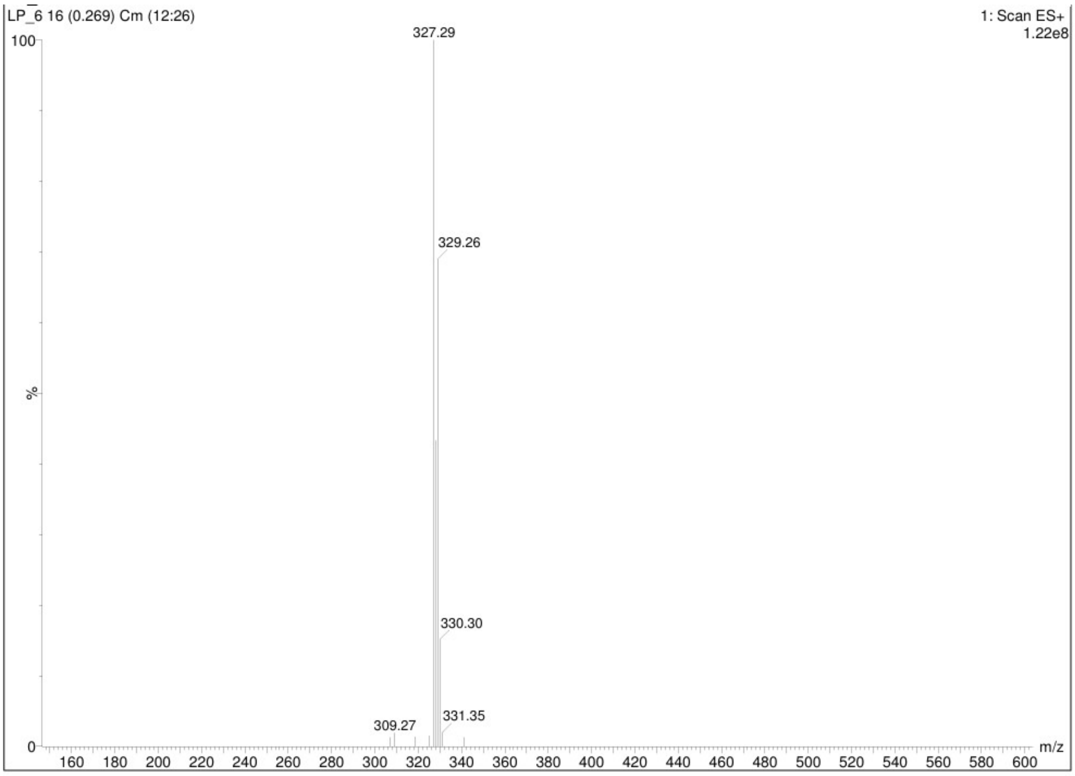

Fig. S23. Mass of **4f**

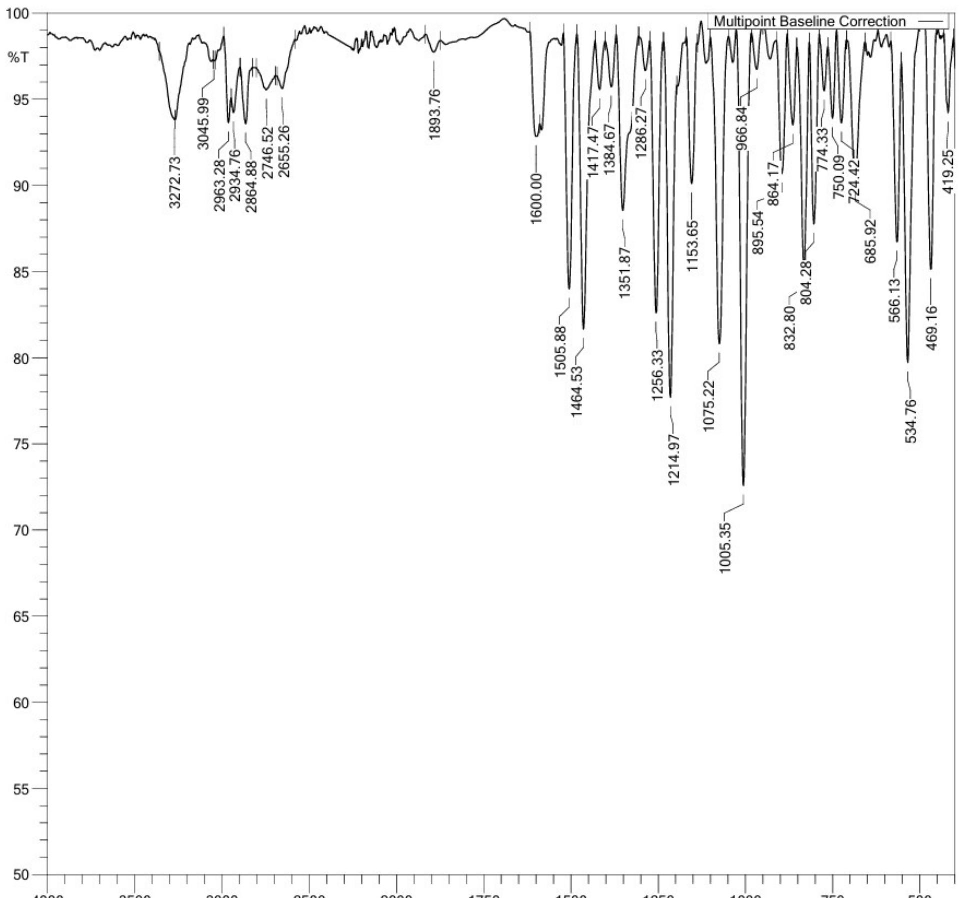

Fig. S24. FTIR of **4f**

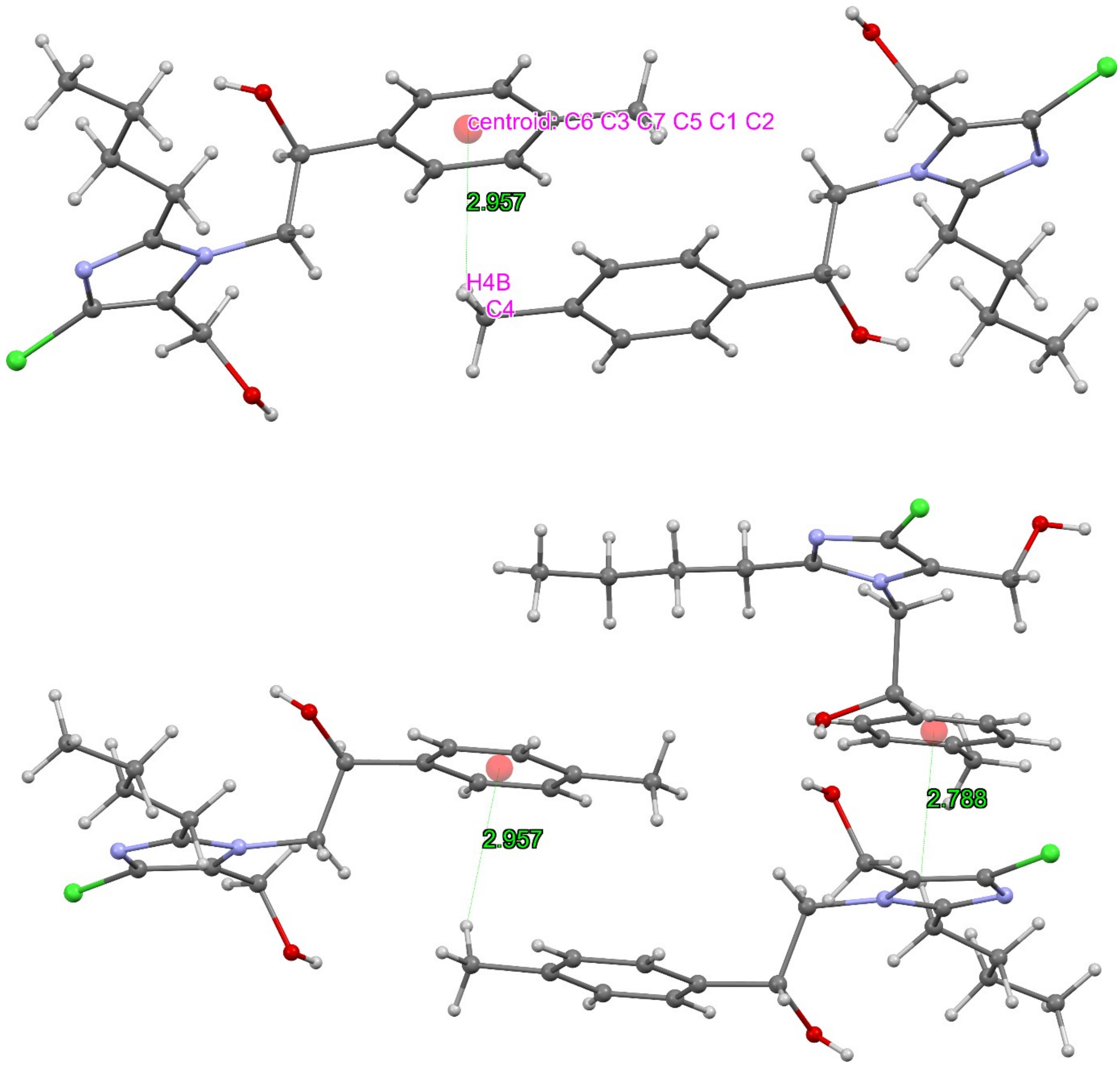

Fig. S25. Parallel stacking interactions formed by the C-H… π interactions

Supplementary Table 1. Cambridge Structural Database (CSD) analysis of bond lengths in molecules related to compound 4c. The table presents bond lengths from 14 crystal structures containing the (2-butyl-4-chloro-1*H*-imidazol-5-yl)methanol fragment. Average bond lengths along with their standard deviations are provided at the end of the table.

CSD RefCode	Cl1-C12	C12-C10	C10-C11	C11-O2	C10-N1	N1-C13	C13-N2	N2-C12	C13-C14	C14-C15	C15-C16	C16-C17
FEFNOU	1.711	1.364	1.429	1.193	1.388	1.344	1.323	1.333	1.474	1.485	1.480	1.439
FEFNUA	1.713	1.371	1.432	1.211	1.390	1.349	1.335	1.342	1.489	1.513	1.509	1.511
HAMRET	1.716	1.373	1.429	1.204	1.389	1.347	1.344	1.330	1.488	1.420	1.577	1.378
ILOPOO	1.723	1.357	1.476	1.416	1.383	1.359	1.327	1.367	1.496	1.489	1.509	1.491
ILOPOO01	1.723	1.357	1.484	1.353	1.386	1.355	1.327	1.36	1.489	1.518	1.515	1.513
ILOPUU	1.712	1.375	1.436	1.215	1.386	1.388	1.346	1.352	1.488	1.528	1.523	1.503
ILOPUU01	1.715	1.367	1.444	1.206	1.382	1.336	1.342	1.351	1.485	1.530	1.523	1.497
KAMPUK	1.717	1.384	1.450	1.218	1.397	1.354	1.340	1.349	1.492	1.527	1.502	1.527
OCAHAC	1.713	1.360	1.492	1.422	1.392	1.362	1.333	1.371	1.495	1.538	1.531	1.524
PAJQUN	1.721	1.349	1.482	1.415	1.388	1.362	1.318	1.365	1.490	1.484	1.453	1.504
QAKXAF	1.716	1.345	1.492	1.401	1.393	1.339	1.335	1.373	1.490	1.517	1.506	1.474
VURTIL	1.694	1.372	1.522	1.440	1.377	1.346	1.328	1.399	1.529	1.508	1.406	1.534
WAKJEY	1.713	1.374	1.435	1.219	1.400	1.351	1.342	1.351	1.491	1.536	1.519	1.515
YALXOZ	1.712	1.348	1.493	1.405	1.389	1.338	1.321	1.380	1.501	1.521	1.520	1.518
**Average**	1.714	1.364	1.464	1.308	1.389	1.352	1.333	1.359	1.493	1.508	1.505	1.495
**Standard Deviation**	0.007	0.012	0.031	0.105	0.006	0.013	0.009	0.019	0.012	0.031	0.039	0.041
**4c**	**1.715**	**1.350**	**1.485**	**1.413**	**1.382**	**1.357**	**1.316**	**1.364**	**1.501**	**1.475**	**1.517**	**1.511**

Supplementary Table 2. Cambridge Structural Database (CSD) analysis of bond angles in molecules related to compound 4c. This table presents bond angle data from 14 crystal structures containing the (2-butyl-4-chloro-1*H*-imidazol-5-yl)methanol fragment. The average bond angles, along with their standard deviations, are summarized at the end of the table.

CSD RefCode	Cl1-C12-C10	C12-C10-C11	C10-C11-O2	C11-C10-N1	C10-N1-C13	N1-C13-N2	C13-N2-C12	N2-C12-Cl1	N2-C12-C10	C12-C10-N1	N1-C13-C14	N2-C13-C14	C13-C14-C15	C14-C15-C16	C15-C16-C17
FEFNOU	125.98	129.68	126.74	126.92	107.53	111.42	104.92	120.99	112.89	103.24	121.84	126.66	117.79	114.91	115.72
FEFNUA	126.53	129.21	127.14	127.34	107.46	111.78	104.27	120.43	113.04	103.45	124.32	123.88	113.23	113.26	113.74
HAMRET	125.79	129.82	127.74	126.76	106.98	112.28	103.63	120.52	113.69	103.42	125.62	122.08	115.91	112.64	112.95
ILOPOO	126.69	132.44	113.19	124.09	108.43	110.91	104.29	120.37	112.95	103.43	124.00	125.05	113.26	114.72	111.30
ILOPOO01	126.81	132.37	115.77	123.93	108.09	110.96	104.63	120.51	112.68	103.63	123.73	125.30	112.54	113.90	111.79
ILOPUU	126.56	132.02	124.10	124.28	108.31	111.31	104.49	121.27	112.16	103.71	124.13	124.56	112.95	114.82	112.62
ILOPUU01	126.65	132.22	124.22	124.04	108.36	111.19	104.43	121.08	112.27	103.74	124.16	124.65	112.95	114.58	113.16
KAMPUK	126.87	128.74	126.46	127.82	107.40	112.03	104.34	120.33	112.79	103.43	124.20	123.77	112.00	114.25	111.81
OCAHAC	126.97	131.75	113.72	124.33	108.48	110.28	105.28	120.89	112.13	103.86	125.57	124.13	115.85	112.37	113.85
PAJQUN	126.66	132.90	113.96	123.61	107.95	111.01	104.51	120.27	113.07	103.45	124.11	124.88	115.54	116.64	113.92
QAKXAF	127.54	133.37	114.40	122.68	108.92	110.06	105.28	120.69	111.77	103.95	125.36	124.57	114.80	112.61	115.10
VURTIL	128.87	128.49	111.79	125.12	106.08	114.74	102.16	119.90	111.23	105.75	121.41	123.77	118.48	114.65	109.20
WAKJEY	126.94	130.09	126.41	126.43	107.55	111.68	104.42	120.20	112.87	103.48	124.73	123.57	115.51	110.94	113.89
YALXOZ	127.48	133.31	113.67	123.31	108.66	111.4[illegible]	104.08	120.08	112.4[illegible]	103.38	123.39	125.18	113.4[illegible]	113.3[illegible]	112.52

						3			3				4	1	
**Average**	126.88	131.17	119.95	125.05	107.87	111.5<br>1	104.34	120.54	112.5<br>7	103.71	124.04	124.43	114.5<br>9	113.8<br>3	112.97
**Standard Deviation**	0.74	1.75	6.52	1.67	0.76	1.11	0.76	0.40	0.62	0.62	1.22	1.06	1.99	1.42	1.65
**4c**	**127.03**	**121.90**	**109.62**	**124.13**	**107.80**	**111.1<br>6**	**104.61**	**120.43**	**112.5<br>2**	**103.90**	**122.09**	**126.71**	**114.8<br>8**	**113.7<br>7**	**112.41**

Supplementary Table 3. Cambridge Structural Database (CSD) analysis of torsion angles in molecules related to compound 4c. The table presents tortion angles of methanol/formaldehyde and butyl side chain from 14 crystal structures.

CSD RefCode	N1-C10-C11-O2	C12-C10-C11-O2	N1-C13-C14-C15	N2-C13-C14-C15	C13-C14-C15-C16	C14-C15-C16-C17
FEFNOU	-4.90	169.65	160.93	-15.55	-176.90	97.57
	-sp	+ap	+ap	-sp	-ap	+ac
FEFNUA	1.24	-178.24	177.42	-4.19	174.28	-176.49
	+sp	-ap	+ap	-sp	+ap	-ap
HAMRET	0.35	179.60	-106.05	75.96	-177.70	-177.24
	+sp	+ap	-ac	sc	ap	ap
ILOPOO	63.87	-133.57	176.40	-1.13	-177.07	178.49
	sc	-ac	+ap	-sp	-ap	+ap
ILOPOO01	-58.42	117.97	-178.05	1.02	177.06	-178.67
	-sc	+ac	-ap	+sp	+ap	-ap
ILOPUU	-0.03	-180.00	-115.62	63.50	61.74	71.00
	-sp	ap	-ac	+sc	+sc	+sc
ILOPUU01	0.25	-179.68	-115.72	63.37	62.23	70.26
	+sp	-ap	-ac	+sc	+sc	+sc
KAMPUK	2.88	-178.72	155.55	-23.98	-175.75	177.48
	+sp	-ap	+ap	-sp	-ap	+ap
OCAHAC	-59.67	123.77	90.17	-91.43	168.18	-67.31
	-sc	ac	ac	-ac	ap	-sc
PAJQUN	-59.98	117.53	83.56	-96.63	60.63	168.88
	-sc	+ac	+sc	-ac	+sc	+ap
QAKXAF	59.41	-120.08	-94.73	84.72	-178.98	168.10
	-sc	-ac	-ac	+sc	-ap	+ap
VURTIL	63.46	-106.03	-167.31	9.16	65.12	175.59
	+sc	-ac	-ap	+sp	+sc	+ap
WAKJEY	1.79	-178.40	74.94	-106.76	175.10	179.02
	+sp	-ap	+sc	-ac	+ap	+ap
YALXOZ	56.84	-122.54	69.90	-109.59	178.51	-175.94
	+sc	-ac	+sc	-ac	+ap	-ap
**4c**	**-73.15**	**103.27**	**-162.71**	**19.51**	**178.96**	**-177.18**
	**-sc**	**+ac**	**-ap**	**+sp**	**+ap**	**-ap**
